# Incorporating additive genetic effects and linkage disequilibrium information to discover gene-environment interactions using BV-LDER-GE

**DOI:** 10.1186/s13059-025-03815-z

**Published:** 2025-10-03

**Authors:** Zihan Dong, Wei Jiang, Jiangnan Shen, Hongyu Li, Yuhan Xie, Andrew T. DeWan, Hongyu Zhao

**Affiliations:** 1 Department of Biostatistics, Yale School of Public Health, 300 George Street, 5 Floor, New Haven, CT 06511 USA; 2 Center for Perinatal, Pediatric and Environmental Epidemiology, Yale School of Public Health, 1 Church Street, 6 Floor, New Haven, CT 06520 USA; 3 Department of Mathematics, University of Texas at Arlington, Arlington, TX 76019 USA; 4 Division of Data Science, University of Texas at Arlington, Arlington, TX 76019 USA; 5 Center for Innovations in Health Informatics, University of Texas at Arlington, Arlington, TX 76019 USA; 6 Department of Chronic Disease Epidemiology, Yale School of Public Health, New Haven, CT USA

**Keywords:** Gene-environment interaction, Genome-wide interaction scan, Genome-wide association study, Genetic covariance, Linkage disequilibrium, Statistical power

## Abstract

Uncovering environmental factors interacting with genetic factors to influence complex traits is important in genetic epidemiology and disease etiology. We introduce BiVariate Linkage-Disequilibrium Eigenvalue Regression for Gene-Environment interactions (BV-LDER-GE), a statistical method that detects the overall contributions of G × E interactions in the genome using summary statistics of complex traits. In comparison to existing methods which either ignore correlations with additive effects or use partial information of linkage disequilibrium (LD), BV-LDER-GE harnesses correlations with additive genetic effects and full LD information to enhance the statistical power to detect genome-scale G × E interactions.

**Supplementary Information:**

The online version contains supplementary material available at 10.1186/s13059-025-03815-z.

## Background

The study of gene-environment interactions (G $\times$ E interactions) is a crucial element of genetic epidemiology, which has garnered significant attention and ongoing debates about its nature for decades [1]. Consequently, numerous study designs and statistical tools have been developed to study G $\times$ E interactions from different perspectives. Based on data from genome-wide association studies (GWAS), genome-wide interaction scan (GWIS) [2] analyzes the variant-by-E interaction effect on the phenotype for each individual single nucleotide polymorphism (SNP). Because the effect size of each individual variant-by-E interaction may be weak, a more powerful way to discover environment factors having interactions with SNPs is to detect the overall contributions of G $\times$ E interactions in the genome.

Currently, there are two distinct approaches to detecting G $\times$ E interactions at the genome level. First, we can use the polygenic risk score (PRS), which is calculated by aggregating the SNP dosages weighted by SNP effects, as a proxy to represent the overall risk of the disease attributed to genetic factors. Then, we can investigate G $\times$ E interactions by regressing the phenotype against the PRS by environment interaction (PRS-by-E) term [3–6]. PIGEON [7], a model that integrates the variant-level additive effects and G × E interaction effects using a random effects model under the polygenic assumption, demonstrates that the regression coefficient of the PRS-by-E term in the linear regression is mathematically equivalent to the genetic covariance between the genetic additive effect and the G $\times$ E interaction effect, which is called G $\times$ E genetic covariance. By utilizing summary statistics and the information of linkage disequilibrium (LD) encoding dependency among SNPs, PIGEON provides an estimate to the G $\times$ E genetic covariance and to detect whether environmental factors interact with genetic predispositions.

On the other hand, variance-component-based methods have been developed to directly estimate the phenotypic variance attributed to G $\times$ E interactions, which is called G $\times$ E interaction proportion in this article. Many methods have been proposed to estimate G $\times$ E interaction proportion utilizing either individual-level data [8–10] or GWIS summary statistics [7, 11, 12]. PIGEON and GxEsum [11] are two summary-statistics-based methods making use of the LD scores, which are diagonal elements of the squared LD matrix. By noting that incorporating the full information of LD can improve the statistical efficiency of estimating variance components, LDER-GE [12] was further proposed to efficiently estimate the G $\times$ E interaction proportion and enhance the power to detect G $\times$ E interaction at the genome level.

Here we extend the PIGEON and LDER-GE framework by utilizing full LD information to allow a more efficient estimate of both G × E genetic covariance and G × E interaction proportion, and an innovative joint test of them. Our framework, named BiVariate Linkage-Disequilibrium Eigenvalue Regression for Gene-Environment interactions (BV-LDER-GE), inputs the summary statistics for both additive genetic effects and G × E interaction effects. The advantage of BV-LDER-GE originates from two aspects: the joint modeling of G × E interaction proportion term and G × E genetic covariance term improves the statistical power to detect G × E interaction effects and incorporating full LD information enhances the model parameter estimation efficiency. We note that different from BV-LDER-GE, LDER-GE only focuses on the phenotypic variance explained by G × E interaction effects using full LD information and summary statistics.

Extensive simulations demonstrate that BV-LDER-GE yields more accurate estimates on G × E genetic covariance and increases power to detect genome-level G × E interaction effects than LDER-GE and PIGEON, while controlling the type-I error rate well. We analyzed 151 environment-phenotype (E-Y) pairs using data from 307,259 unrelated European ancestry subjects in the UK Biobank [13]. After Bonferroni correction, BV-LDER-GE detected 63 statistically significant signals on the genome-level G × E interactions, while LDER-GE identified 35 and PIGEON identified 25. For the statistical detection of G × E genetic covariance, BV-LDER-GE identified 50 signals and PIGEON identified 40 signals, which were all covered by BV-LDER-GE. These real data results suggest the widespread presence of G × E interaction effects on human complex traits across commonly studied environmental covariates, and position BV-LDER-GE as a potential tool for prioritizing targets in more detailed future G × E studies.

## Results

### Model overview

We consider the following model to incorporate both additive and interaction effects:

1 $${Y}_{i}={\sum }_{j=1}^{M}{G}_{ji}{\beta }_{j}+{\sum }_{j=1}^{M}{S}_{ji}{\gamma }_{j}+{\epsilon }_{1i}{E}_{i}+{\epsilon }_{0i},$$

where ${Y}_{i}$ is the standardized phenotype adjusting the fixed effects of covariates and ${E}_{i}$ is the standardized environment variable for subject *i*,

2 $$\left[\begin{array}{c}{\beta }_{j} \\ {\gamma }_{j}\end{array}\right]\sim N([\begin{array}{c}0\\ 0\end{array}], [\begin{array}{cc}{h}_{g}^{2}/M& {\rho }_{IG}/M\\ {\rho }_{IG}/M& {h}_{I}^{2}/M\end{array}]),$$

where ${\beta }_{j}$ is the additive genetic effect size for variant *j*, and ${\gamma }_{j}$ is the G × E interaction effect size for variant *j*, ${h}_{g}^{2}$ is the narrow-sense heritability defined as the phenotypic variance explained by additive genetic effects, ${h}_{I}^{2}$ is the G × E interaction proportion, and ${\rho }_{IG}=\sqrt{{h}_{g}^{2}{h}_{I}^{2}}{r}_{IG}$ is the G × E genetic covariance between ${\beta }_{j}$ and ${\gamma }_{j}$. Here we use ${r}_{IG}$ to represent the G × E genetic correlation. For the residual term, we model:

3 $$[\begin{array}{c}{\epsilon }_{0} \\ {\epsilon }_{1}\end{array}]\sim N([\begin{array}{c}0\\ 0\end{array}], [\begin{array}{cc}{\sigma }_{0}^{2}& {\rho }_{01}\\ {\rho }_{01}& {\sigma }_{1}^{2}\end{array}]),$$

where ${\epsilon }_{1}$ is the residual with an interaction effect with the environmental factors and has variance ${\sigma }_{1}^{2}$, ${\epsilon }_{0}$ is the residual without an interactive effect with the environmental factors and has variance ${\sigma }_{0}^{2}$, and ${\rho }_{01}$ is the covariance between ${\epsilon }_{1}$ and ${\epsilon }_{0}$. We assume that the additive genetic effects and G × E interaction effects are polygenic. Besides, the environment variable is either non-heritable or polygenically heritable in which the additive genetic effects are uncorrelated with the G × E interaction effects on the phenotype.

### Joint modeling of G × E interaction proportion and G × E genetic covariance

**Fig. 1:**
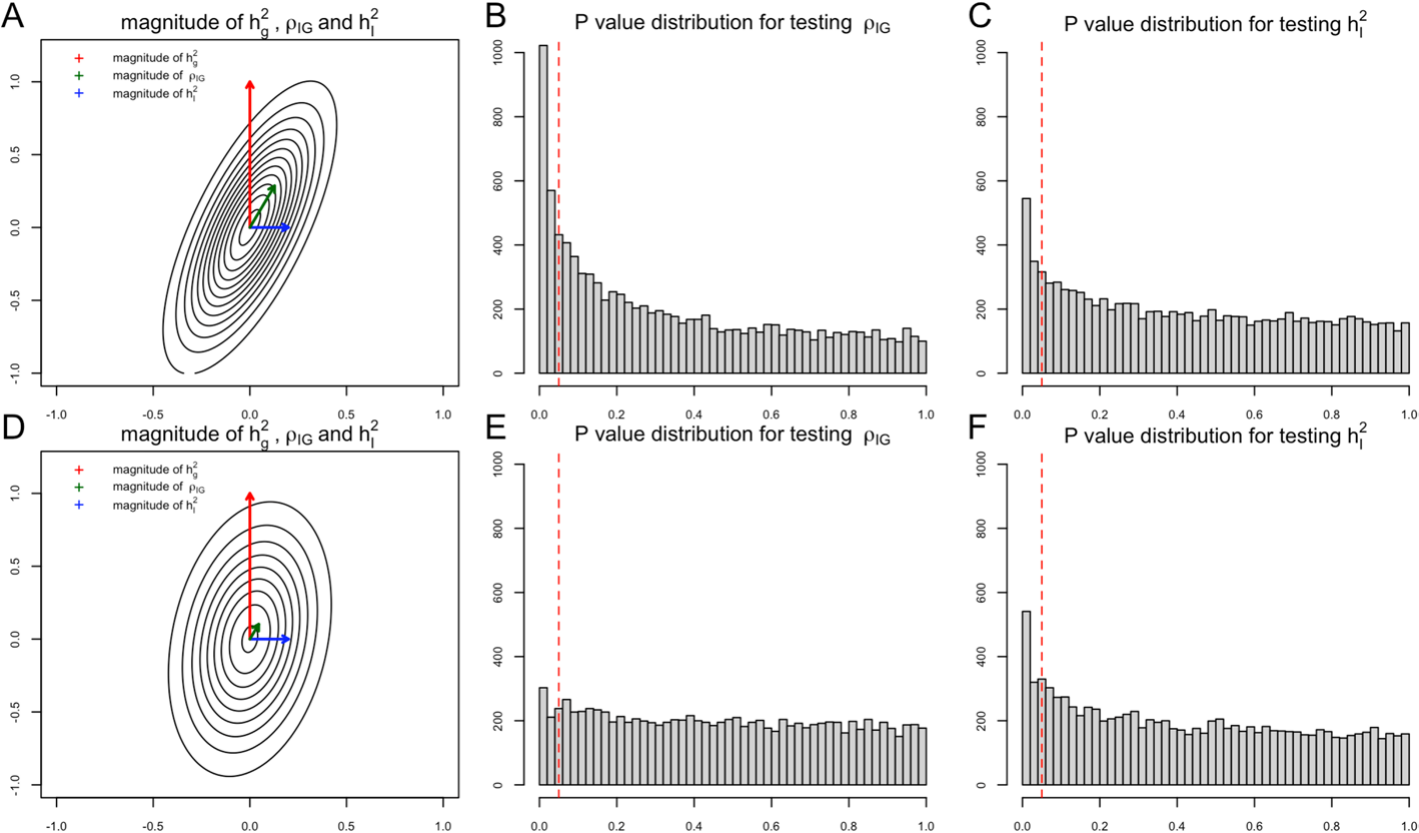
An illustration of the relationship between statistical power and magnitude of ${h}_{I}^{2}$ and ${r}_{IG}$

**Fig. 2:**
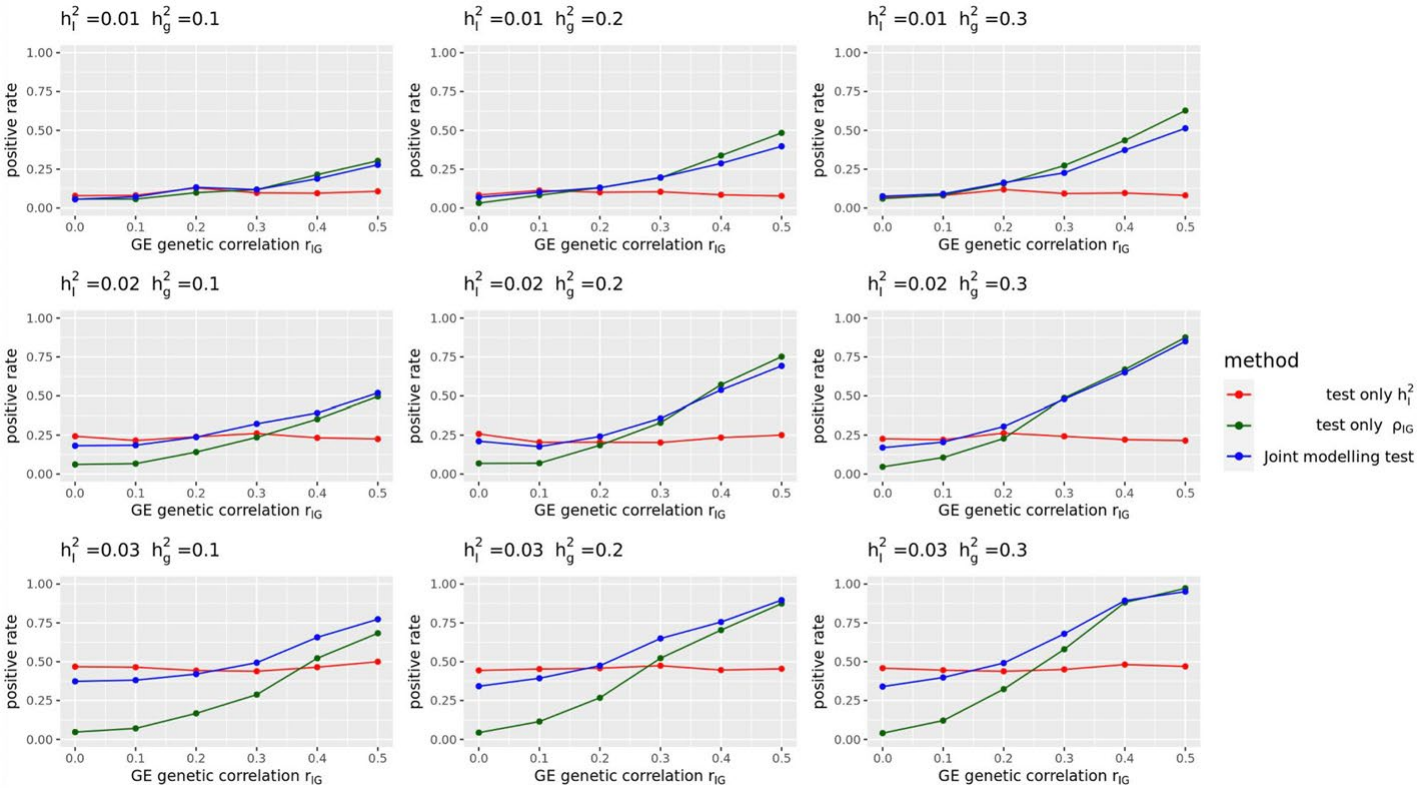
Statistical power of different GE testing methods of LDER-GE-based in simulation studies with various parameters

**Fig. 3:**
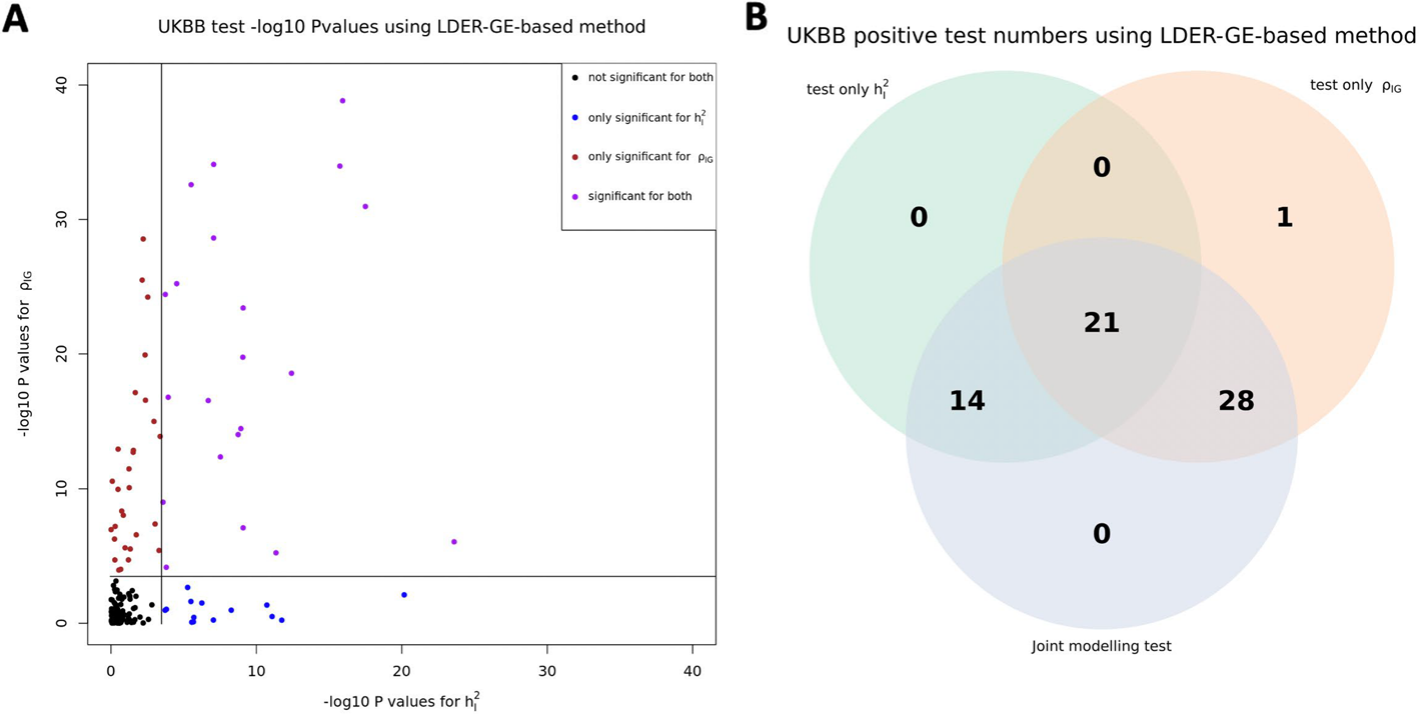
UKBB real data analysis results for the 151 E-Y pairs

The final goal of our first task is to detect the existence of G × E interaction proportion ${h}_{I}^{2}$. Note that in a well-defined variance–covariance matrix, if one off-diagonal entry is non-zero, then it implies that its corresponding two diagonal entries are both non-zero. In the genetic random effects model, a non-zero G × E genetic covariance term ${\rho }_{IG}$ implies that both ${h}_{I}^{2}$ and ${h}_{g}^{2}$ are non-zero. Generally, the narrow-sense heritability ${h}_{g}^{2}$ has much larger magnitude than G × E interaction proportion [14], and ${\rho }_{IG}$ contains partial statistical signal from narrow-sense heritability when the G × E genetic correlation ${r}_{IG}$ is non-zero (Fig. 1). Depending on the magnitude of ${h}_{g}^{2}$, ${h}_{I}^{2}$, and ${r}_{IG}$, the statistical evidence of ${\rho }_{IG}$ is even stronger than ${h}_{I}^{2}$ in some scenarios (Fig. 1), either in simulation studies (Fig. 2) or real data analysis (Fig. 3). Several statistical methods with input of genetic summary statistics data have been developed to estimate ${h}_{I}^{2}$, ${h}_{g}^{2}$, and ${\rho }_{IG}$ [7, 11, 12, 15–17]. For joint modeling, we write:

4 $$\widehat{{\varvec{V}}}=[\begin{array}{c}\widehat{{h}_{I}^{2}} \\ \widehat{{\rho }_{IG}}\end{array}]\sim N([\begin{array}{c}{h}_{I}^{2}\\ {\rho }_{IG}\end{array}], \boldsymbol{\Sigma }),$$

where $\boldsymbol{\Sigma }$ is the true variance–covariance matrix for the two estimates. Here we plug in the empirical variance–covariance matrix $\widehat{\boldsymbol{\Sigma }}$ obtained via delete-block-wise jackknife. In order to indirectly test ${h}_{I}^{2}$, we propose a joint modeling test, to test the BV vector $$\left[\begin{array}{c}{h}_{I}^{2}\\ {\rho }_{IG}\end{array}\right]=\left[\begin{array}{c}0\\ 0\end{array}\right]$$, which is equivalent to testing the squared Mahalanobis distance estimated by $\widehat{{d}^{2}}={\widehat{\boldsymbol{ }{\varvec{V}}}}^{{\varvec{T}}}{\widehat{\boldsymbol{\Sigma }}}^{-1}\widehat{{\varvec{V}}}$.

For simulation studies using real UKBB genotype data, we have a total of 54 different parameter settings, and none of the three methods dominates the other two across all parameter settings (Fig. 2). For example, when ${r}_{IG}$ is very low or equal to 0, testing only ${h}_{I}^{2}$ achieves the highest power among all three methods. On the contrary, as all parameters ${h}_{g}^{2}$, ${h}_{I}^{2}$, and ${r}_{IG}$ increase, testing only ${\rho }_{IG}$ starts to achieve higher power. And joint modeling test achieves a balance of two single-variate test methods and works the best when the true ${h}_{I}^{2}$ term is very small but ${h}_{g}^{2}$ and ${r}_{IG}$ terms are relatively large (Fig. 2). We also evaluated the type-I error rate for three tests and concluded that all three methods control the type-I error rate well (Table 1), whether they test ${h}_{I}^{2}$ directly or indirectly. Since theoretically no optimal test exists for the G × E interaction testing task across all scenarios, we applied our method to UKBB real data analysis (Methods), and the results demonstrated the efficacy of the joint modeling test (Fig. 3). Across the 151 E-Y pairs tested, there are more statistically strong (− log(*p* value) > 10) signals for ${\rho }_{IG}$ than ${h}_{I}^{2}$ (Fig. 3A), which can be partially attributed to the larger narrow-sense heritability of the phenotypes. Overall, testing only ${h}_{I}^{2}$ identified 35 signals, and testing only ${\rho }_{IG}$ identified 50 signals, while joint modeling test identified 63 signals, covering all signals found by the other two methods except one signal from ${\rho }_{IG}$ (Fig. 3B). A full list for all parameter estimates and *p* values can be found in Additional file 3: Table S1.

**Table 1:** Type-I error rate at 0.05 level for three LDER-GE-based methods to test ${h}_{I}^{2}$, each scenario with 500 replications

Method	Narrow-sense heritability ${h}_{g}^{2}$	Type-I error rate
Test only ${h}_{I}^{2}$	0.1	0.045
	0.2	0.048
	0.3	0.054
	Average	0.049
Test only ${\rho }_{IG}$	0.1	0.055
	0.2	0.051
	0.3	0.051
	Average	0.052
Joint modeling test	0.1	0.052
	0.2	0.050
	0.3	0.047
	Average	0.050

### Utilize full LD information to estimate G × E genetic covariance more accurately

We have derived the following moment condition for the product of additive genetic effect (GWAS) association vector and G × E interaction effect (GWIS) association vector (Supplementary Note 1):

5 $$E\left({{\varvec{Z}}}_{{\varvec{G}}}{{\varvec{Z}}}_{{\varvec{I}}}^{{\varvec{T}}}\right)=\sqrt{{N}_{G}{N}_{I}}{\rho }_{IG}{\varvec{L}}/M+2{N}_{S}\left({\rho }_{IG}+{\rho }_{01}\right){\varvec{R}}/\sqrt{{N}_{G}{N}_{I}},$$

where ${{\varvec{Z}}}_{{\varvec{G}}}$ is the Z-score vector for GWAS with sample size ${N}_{G}$, and ${{\varvec{Z}}}_{{\varvec{I}}}$ is the Z-score vector for G × E interaction effect with sample size ${N}_{I}$. Here ${N}_{S}$ is the sample overlap of two association analysis, ${\varvec{R}}$ is the LD matrix for all variants, and ${\varvec{L}}={{\varvec{R}}}^{{\varvec{T}}}{\varvec{R}}$ is the LD score matrix. We conducted eigen-decomposition on LD matrix as ${\varvec{R}}={\varvec{U}}{\varvec{D}}{{\varvec{U}}}^{T}$, where ${\varvec{D}}$ has diagonal eigenvalues and ${\varvec{U}}$ has eigenvectors and is orthogonal. We transform the original Z-score vectors as ${\widetilde{{\varvec{Z}}}}_{{\varvec{G}}}^{\boldsymbol{ }}={{\varvec{D}}}^{\left(-1/2\right)}{{\varvec{U}}}^{{\varvec{T}}}{{\varvec{Z}}}_{{\varvec{G}}}$ and ${\widetilde{{\varvec{Z}}}}_{{\varvec{I}}}^{\boldsymbol{ }}={{\varvec{D}}}^{\left(-1/2\right)}{{\varvec{U}}}^{{\varvec{T}}}{{\varvec{Z}}}_{{\varvec{I}}}$ and obtain the moment condition of the *j*th diagonal element of the transformed vector product:

6 $$E{\left({\widetilde{{\varvec{Z}}}}_{{\varvec{G}}}^{\boldsymbol{ }}{\widetilde{{\varvec{Z}}}}_{{\varvec{I}}}^{\boldsymbol{ }{\varvec{T}}}\right)}_{j,j}=\sqrt{{N}_{G}{N}_{I}}{\rho }_{IG}{D}_{j,j}/M+\frac{2{N}_{S}\left({\rho }_{IG}+{\rho }_{01}\right)}{\sqrt{{N}_{G}{N}_{I}}}.$$

The eigen-decomposition and the subsequent Z-vector transformation enable us to exploit more genetic architecture information and result in higher statistical efficiency in estimation.

Formulas (5) and (6) indicate that sample overlap between GWAS and GWIS does not affect the parameter estimates of ${{\varvec{\rho}}}_{{\varvec{I}}{\varvec{G}}}^{ }$ as ${{\varvec{N}}}_{{\varvec{S}}}$ appears only in the intercept term. This means that the GWAS and GWIS datasets can be identical, separate, or partially overlapping. In the UKBB application, the same set of individuals was used to compute both ${{\varvec{Z}}}_{{\varvec{G}}}$ and ${{\varvec{Z}}}_{{\varvec{I}}}$, consistent with this property.

**Fig. 4:**
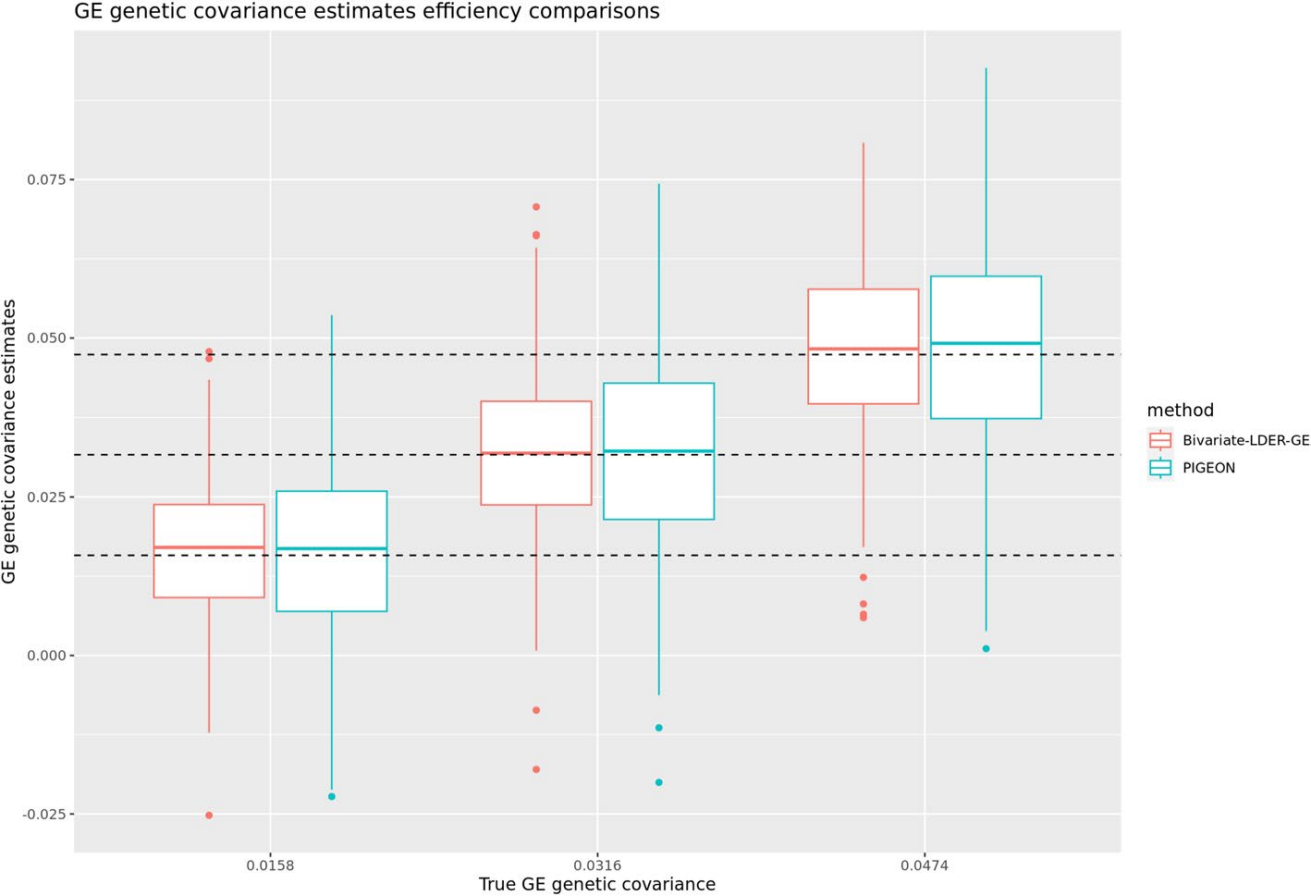
Statistical efficiency comparison of GE genetic covariance estimation for Bivariate-LDER-GE and PIGEON in simulations using real UKBB genotype panel

Through simulations, we show that the empirical standard deviation and root mean square rate of BV-LDER-GE estimate is consistently lower than PIGEON across all simulation scenarios. The average empirical standard deviation across all simulation scenarios of BV-LDER-GE is 23.7% less than PIGEON, approximately equivalent to a sample size increase of 53% (Additional file 3: Table S2). The root mean squared error of BV-LDER-GE is also consistently lower than PIGEON across all simulation scenarios (Additional file 3: Table S2). We selected three simulation scenarios ${(h}_{I}^{2}{=0.01, h}_{g}^{2}{=0.1,r}_{IG}=0.5),{(h}_{I}^{2}{=0.02, h}_{g}^{2}{=0.2,r}_{IG}=0.5)$, and ${(h}_{I}^{2}{=0.03, h}_{g}^{2}{=0.3,r}_{IG}=0.5)$ to visualize the estimates distribution, statistical efficiency, and unbiasedness (Fig. 4). For UKBB real data, BV-LDER-GE detected G × E genetic covariance on 50 E-Y pairs while PIGEON only identified 40. For the 151 E-Y pairs, the average reported standard error of BV-LDER-GE for the 151 E-Y pairs was 30.8% lower than PIGEON (Additional file 3: Table S2), larger but consistent with simulation results.

The authors of PIGEON [7] showed that assuming perfectly fitted PRS, the expected value of the coefficient of the PRS-by-E term in a linear regression model equals to the ratio of G × E genetic covariance and the narrow-sense heritability of the phenotype:

$$Y={\beta }_{1}PRS+{\beta }_{2}E+{\beta }_{3}PRS*E+\upepsilon ,$$

$$E\left(\widehat{{\beta }_{{3}_{\text{lm}}}}\right)=\frac{{\rho }_{GE}}{{h}_{g}^{2}}.$$

**Fig. 5:**
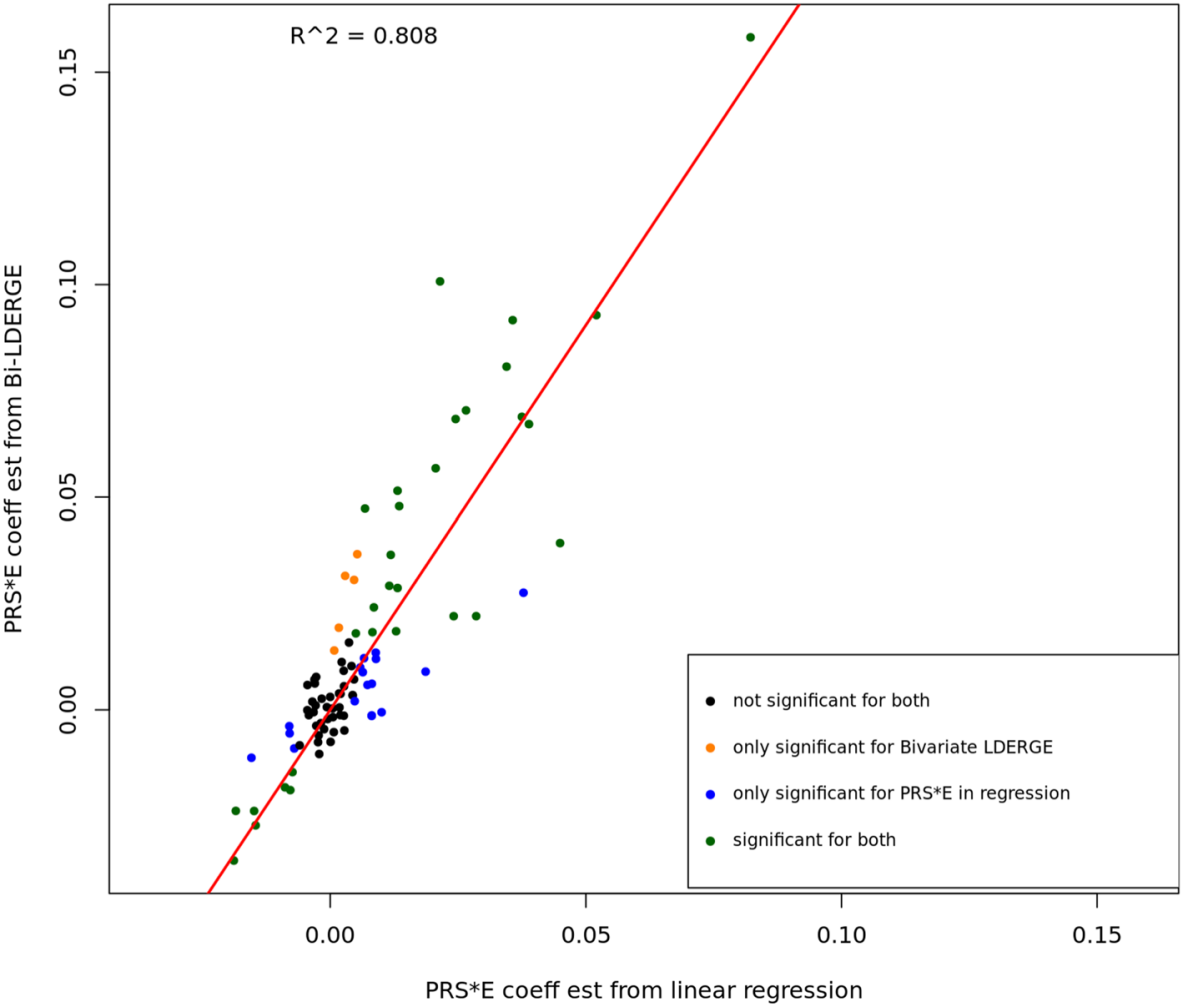
The PRS-by-E effect coefficient estimates from Bivariate-LDER-GE and linear regression

We conducted linear regression analysis on UKBB subjects using fitted PRS from external GWAS summary statistics on the 13 phenotypes and the seven environment covariates to compare the PRS-by-E regression coefficients and G × E genetic covariance estimates using BV-LDER-GE. The PRS fitting was conducted using SDPR [18]. The details for fitting PRS, linear regression, and coefficients comparison can be found in Supplementary Note 2. As shown in Fig. 5, the estimates are highly correlated (*R*^2^ = 0.808) and in all E-Y pairs where both methods are significant, the signs of PRS-by-E effects are consistent. This demonstrates the reliability of using additive genetic effect and G × E interaction summary statistics to study the PRS-by-E effect on the phenotype. Note that the estimates of the PRS-by-E regression coefficient is smaller in magnitude than estimated from BV-LDER-GE (Fig. 5, Additional file 3: Table S3) due to the attenuation bias resulted from imperfectly fitted PRS [7, 19] with measurement error. Our results indicate the potential of a more convenient and feasible alternative to evaluate the PRS-by-E effect without fitting PRS [7]. On the other hand, the *p* value of PRS-by-E regression test can be substantially lower than BV-LDER-GE (Additional file 3: Table S3), because BV-LDER-GE only utilizes summary-level statistics instead of individual-level genotype data. Such PRS-by-E effect direction and magnitude analysis may provide biological interpretation on how the environmental and genetic factors shape the outcome together.

Some of our G × E interaction effect conclusions are consistent with the literature. For example, type 2 diabetes (T2D) has significant marginal PRS-by-E signals on all seven environmental covariates studies. Among them, the PRS-by-E interaction effect with sex [20, 21] and smoking [4, 22] has been reported. Meanwhile, we also report novel interaction discoveries: increased age, being a female, and having higher BMI and drinking frequency magnify the genetic effect of T2D in the European population represented by UKBB. We further ran a conditional PRS-by-E regression including all seven environmental covariates and their PRS-by-E terms in one regression model. Pm2.5 and Townsend deprivation index became nonsignificant while the other five environmental covariates remained significant with multiple testing correction (Additional file 3: Table S4). This demonstrates that multiple environmental exposures and health-related factors interact with genetic predisposition of T2D risk, which might inspire future research direction to the mechanism and intervention regarding T2D and related diseases.

### Correct the confounding effects introduced by heritable environmental variable

**Fig. 6:**
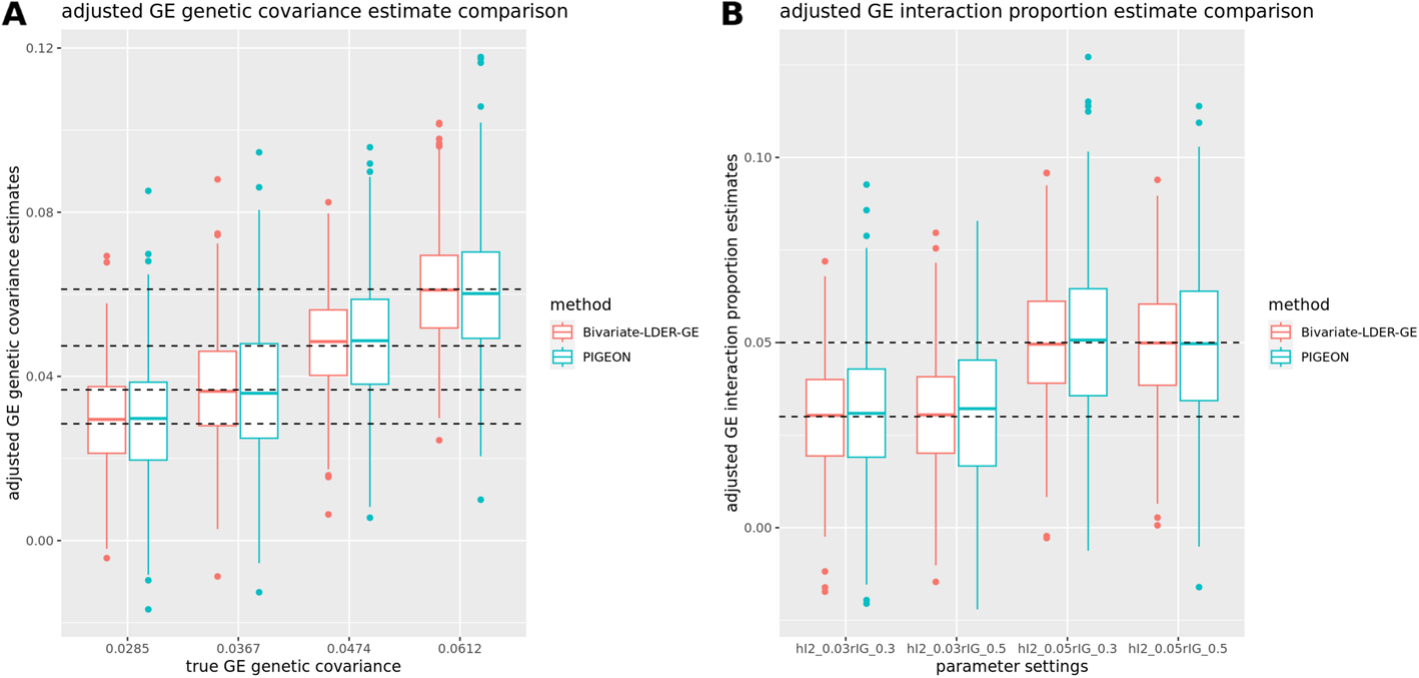
Statistical efficiency comparison of adjusted estimation of GE interaction proportion and GE genetic covariance with heritable environmental covariate confounding effects

One important assumption about the model is that the environment variable is either non-heritable or is polygenically heritable in which its additive genetic effects are uncorrelated with the G × E interaction effects on the phenotype. Many other studies consider heritable phenotypes as the interactive environmental variable [7, 23], such as BMI and alcohol intake [11, 12], and there is a need to test and correct for the potential confounding effect. PIGEON [7] developed a model and framework on this, but utilized only partial LD information through LD score regression [15]. And we improve the existing method for more accurate correction using full LD information. We write the heritable E as

7 $${E}_{i}={\sum }_{j=1}^{M}{G}_{ji}{\alpha }_{j}+{\epsilon }_{2},$$

the genetic random effects as

8 $$[ \begin{array}{c}{\beta }_{j}\\ {\gamma }_{j}\\ {\alpha }_{j}\end{array} ]{\sim }^{iid} N ( [ \begin{array}{c}0\\ 0\\ 0\end{array} ], [ \begin{array}{ccc}{h}_{g}^{2}/M& {\rho }_{IG}/M& {\rho }_{IE}/M\\ {\rho }_{IG}/M& {h}_{I}^{2}/M& {\rho }_{GE}/M\\ {\rho }_{IE}/M& {\rho }_{GE}/M& {h}_{E}^{2}/M\end{array} ] ),$$

and the moment condition for the transformed Z-score product (Supplementary Note 1),

9 $$E{\left({\widetilde{{\varvec{Z}}}}_{{\varvec{E}}}^{\boldsymbol{ }}{\widetilde{{\varvec{Z}}}}_{{\varvec{I}}}^{\boldsymbol{ }{\varvec{T}}}\right)}_{j,j}=\sqrt{{N}_{E}{N}_{I}}\left({\rho }_{IE}\left(1+{h}_{E}^{2}\right)\right){D}_{j,j}/M+{c}_{1},$$

10 $$E{\left({\widetilde{{\varvec{Z}}}}_{{\varvec{I}}}^{\boldsymbol{ }}{\widetilde{{\varvec{Z}}}}_{{\varvec{I}}}^{\boldsymbol{ }{\varvec{T}}}\right)}_{j,j}={N}_{I}\left({h}_{I}^{2}+2{{\rho }_{IE}}^{2}\right){D}_{j,j}/M+{c}_{2},$$

11 $$E{\left({\widetilde{{\varvec{Z}}}}_{{\varvec{G}}}^{\boldsymbol{ }}{\widetilde{{\varvec{Z}}}}_{{\varvec{I}}}^{\boldsymbol{ }{\varvec{T}}}\right)}_{j,j}=\sqrt{{N}_{G}{N}_{I}}{(\rho }_{IG}+{\rho }_{GE}{\rho }_{IE}){D}_{j,j}/M+{c}_{3},$$

where ${\widetilde{{\varvec{Z}}}}_{{\varvec{E}}}^{\boldsymbol{ }}={{\varvec{D}}}^{\left(-1/2\right)}{{\varvec{U}}}^{{\varvec{T}}}{{\varvec{Z}}}_{{\varvec{E}}}$ is the transformed Z-score vector for the GWAS on E, with sample size${N}_{E}$, and ${c}_{s}$ are the simplified intercept terms. Identifying the slope coefficients of Eqs. (9)– (11), we iteratively estimate${h}_{E}^{2}, {\rho }_{IE}, {h}_{I}^{2}, {\rho }_{GE}$, and ${\rho }_{IG}$ and use delete-block wise jackknife for inference. We finally use the corrected ${h}_{I}^{2}$ and ${\rho }_{IG}$ to conduct the joint modeling test on the genome-level G × E interaction proportion or estimate its magnitude directly. Our simulations showed that both BV-LDER-GE and PIGEON produce unbiased corrected estimation for G × E interaction proportion and G × E genetic covariance, but the estimation efficiency of BV-LDER-GE was 22.2% higher than PIGEON for G × E interaction proportion and 24.7% for G × E genetic covariance (Fig. 6, Additional file 3: Table S5).

**Fig. 7:**
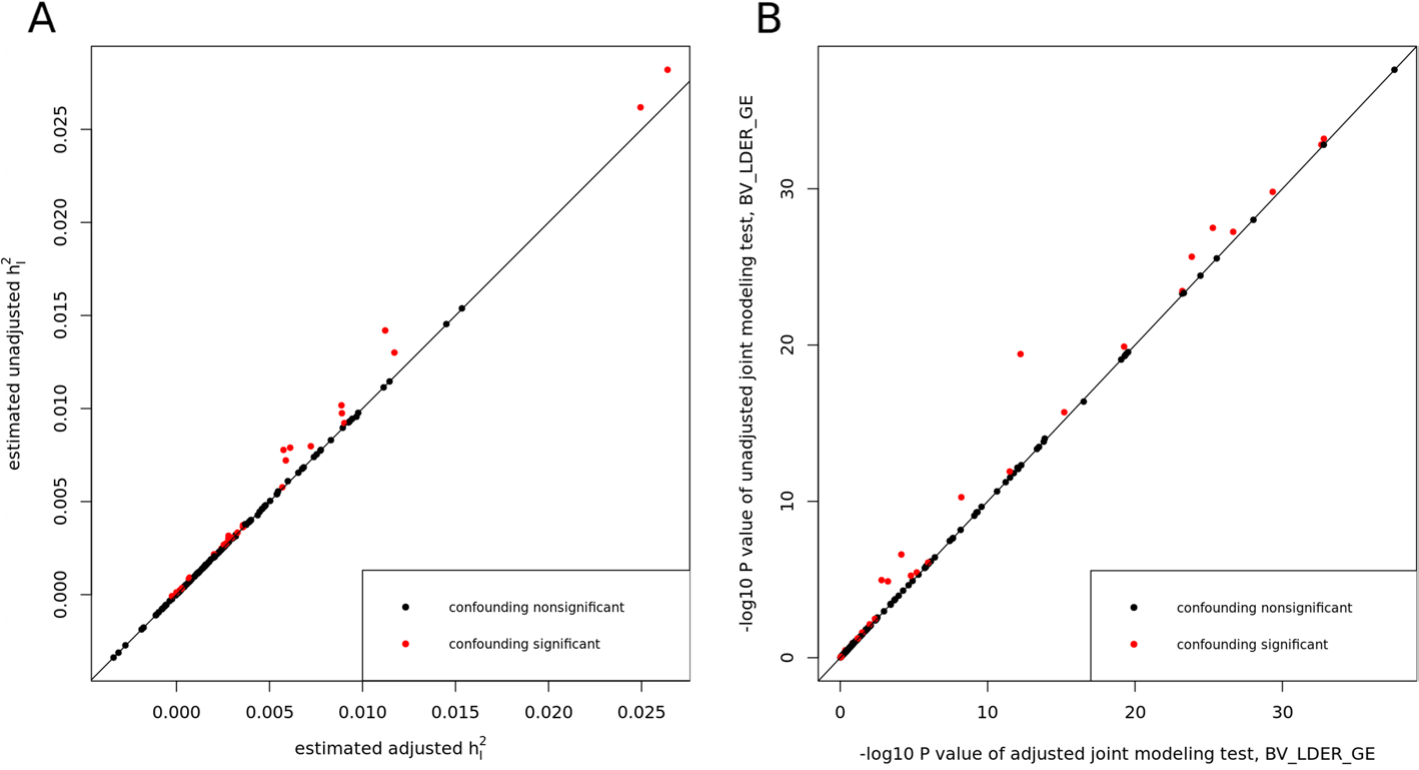
Adjusting for genetic effect of E for the 151 E-Y pairs in UKBB using BV-LDER-GE

We applied two debiased methods to the 151 E-Y pairs of UKBB dataset. Through the 151 tests, BV-LDER-GE identified 30 pairs with G × E interaction proportion bias introduced by the genetic components of the environmental variables, covering all 19 pairs of PIGEON. The confounding test is equivalent to testing term ${\rho }_{IE}$ (Eq. (10)). After adjusting for potential confounding effects, the BMI-CHO and BMI-LDL pairs failed to pass the joint modeling test of BV-LDER-GE and the rest 61 positive unadjusted BV-LDER-GE joint modeling test remained significant. The details of these 151 adjusted E-Y pairs can be found in Additional file 3: Table S6. As Fig. 7A shows, the adjusted ${h}_{I}^{2}$ estimates are always smaller than the unadjusted estimates, consistent with the theoretical derivation (Eq. (10)). And the *p* values will increase if the confounding effect is non-trivial (Fig. 7B). For common practice, we recommend testing the existence of term ${\rho }_{IE}$ first as some social economic status covariates may still contain genetic components [24]. If the test is significant then an adjustment of genetic confounding effect should be considered, even if a significant confounding effect may still lead to small bias (Fig. 7A).

## Discussion

In this study, we present BV-LDER-GE to tackle the challenges in gene-environment interaction research for human complex traits. We propose a novel joint modeling test which gathers statistical signal from both G × E interaction proportion and G × E genetic covariance to test genome-level G × E interaction effects more powerfully. The joint modeling test leverages complete information from G × E interaction signals while also draws on partial information from additive genetic effects. Our simulation studies reveal that the joint modeling test does not consistently outperform the direct tests for either G × E interaction proportion or G × E genetic covariance across the entire parameter space, as shown in Fig. 2. However, UKBB real data analysis showed that the joint modeling test almost perfectly captured all the signals from both direct tests on GE proportion and G × E genetic covariance, except for one E-Y pair (Fig. 3B). The improved statistical power results from a well-balanced trade-off between the two components to be captured by the joint modeling test, where even if the power of the joint modeling test is somewhat diluted by a weaker signal from one component, the loss in power remains minimal (Fig. 2, x-axis, ${r}_{IG}$ ≤ 0.2). However, in such circumstance, the power of the joint modeling test is still substantially higher than the power to test the weaker component alone. Simulations showed that the joint modeling test has well-controlled type-I error rate (Table 1), suggesting that the empirical estimation of the variance–covariance matrix of the estimates, as computed via the delete-block-wise jackknife method (Eq. (4)), is reasonable and approximates the true distribution accurately.

One limitation of the joint modeling test is that it only reports a *p* value due to the limited interpretability of an output squared Mahalanobis distance. For those needing detailed parameter estimates of G × E interaction proportion or G × E genetic covariance, direct tests are necessary, albeit potentially less powerful than the joint modeling approach. Moreover, individual-level-based estimation tools [8, 9, 25, 26], which can produce more accurate estimates of G × E interaction proportion, come at the expense of higher computational resources and increased memory demands. Acknowledging the benefits and drawback, the joint modeling test and BV-LDER-GE framework are recommended for identifying G × E interaction effects and prioritizing targets in future G × E studies. Based on both our simulation and real data results, we recommend that users first apply the joint Mahalanobis test to assess the overall magnitude of the GE interaction. If the joint test is significant, users can then estimate ${{\varvec{h}}}_{{\varvec{I}}}^{2}$ and ${{\varvec{\rho}}}_{{\varvec{I}}{\varvec{G}}}$ separately to gain more detailed biological insights. We advise against testing ${{\varvec{h}}}_{{\varvec{I}}}^{2}$ and ${{\varvec{\rho}}}_{{\varvec{I}}{\varvec{G}}}$ when the joint test is nonsignificant, to control the type-I error rate.

The second contribution of BV-LDER-GE is more accurate parameter estimation utilizing full LD information. BV-LDER-GE builds on the PIGEON framework [7], which introduced the concept of G × E genetic covariance alongside the proposition of random effect modeling for genetic additive and G × E interaction effects. Through extensive simulations, we illustrate the statistical efficiency improvement of BV-LDER-GE via using more LD information. The average efficiency improvement for UKBB data analysis across the 151 E-Y pairs is close the average efficiency improvement of the simulations, but this does not necessarily reflect any specific E-Y pair due to different and largely undetermined genetic architecture, effect sizes, and effective SNP sparsity. As discussed [7], the estimated G × E genetic covariance and narrow-sense heritability can be employed to model the PRS-by-E effect on the outcome phenotype in a linear model, without fitting the PRS practically. This summary statistics approach offers a distinct advantage over the PRS-by-E regression method, which is susceptible to attenuation bias [26] due to imperfect PRS fitting and is robust against sample overlap between the target (GWIS) and base (GWAS) samples [7]. Furthermore, the equivalence relationship underscored here enhances both the interpretability and biological relevance of studying G × E genetic covariance.

One focus of PIGEON work [7] is the hypothesis-free test, which does not presuppose the PRS and outcome share the same phenotype. Rather, it accommodates the PRS of one phenotype and the outcome of another (i.e., GWAS of variable1 and GWIS of variable2), as some PRS-by-E studies [27]. In this study, however, we focused solely on hypothesis-driven analysis where the same phenotype is targeted for both PRS and outcome. Despite this, BV-LDER-GE is well-suited for hypothesis-free analysis, and we anticipate that it would retain higher statistical efficiency in such applications.

BV-LDER-GE also extends PIGEON model to cross-variant product moment conditions for enhancing the detection and correction for the confounding effect when the environmental variable possesses a genetic component. We illustrated the efficiency improvement through simulations and real data analysis. Analyzing 22 phenotypes and three heritable environmental variables, we found that although some confounding effects were significantly detected (see Supplementary Tables 1 and 3), the impact on the G × E interaction proportion generally remains trivial. We recommend proactive detection and correction for confounding effects associated with heritable environmental variables whenever GWAS summary statistics for such variables are available. It is important to note that the presence of a genetic component in environmental variables is a necessary but not sufficient condition for bias [7, 28]. For studies aimed solely at detecting the existence of G × E interaction proportion using either direct test on ${h}_{I}^{2}$ or ${r}_{IG}$ or employing joint modeling test should not theoretically inflate the type-I error rate, as ${\rho }_{IE}$ (Eqs. (10) and (11)) constantly equals to zero under the null hypothesis.

One limitation of our study is the assumption of polygenicity for both the additive genetic effect and the G × E interaction effect. Departures from an independent Gaussian effect-size distribution, including sparsity, will diminish estimation and statistical efficiency [29, 30]. Higher sparsity lowers statistical efficiency but does not affect unbiasedness or inflate the type-I error rate. In contrast, violations of the Gaussian assumption can bias parameter estimates, as the derived expected second moment depends on Gaussianity. Nevertheless, the type-I error rate remains controlled even under such violations, because any distributional assumption collapses to zero under the null hypothesis (no GE interaction). Additionally, as mentioned, the joint modeling test does not report any parameter estimation but only one *p* value for the test. Furthermore, BV-LDER-GE, like any LD-dependent summary statistics framework, struggles to handle rare variants effectively due to their low linkage disequilibrium [31], leading to their exclusion from our “genome-level” G × E interaction results. Another limitation of our approach is that it operates at the genome level rather than the SNP level, even though SNP-level summary statistics are used as input. This design choice allows us to fully leverage genome-wide LD information and additive genetic effects, thereby increasing power for detecting GE interactions. However, high-resolution localization of interaction signals at individual variants would require additional follow-up analyses using GWAS or GWIS results. Our study focuses on common variants, with a relatively fixed SNP count for biobank-scale datasets. Analyses incorporating a much larger set of variants, such as rare variants, would require addressing sparsity and scalability challenges, which we leave for future work.

In conclusion, the success of BV-LDER-GE comes from a dual standpoint: the joint modeling test gathers partial information from additive genetic effect, whose variant-level magnitude is generally stronger than the G × E interaction effect [14]; and the exploit of full LD information enhances the estimation quality of model parameters and subsequent testing. We demonstrated the superiority of BV-LDER-GE via both simulation and real data analysis using UKBB. As a result, BV-LDER-GE offers a valuable tool for prioritization and analysis, adeptly addressing various challenges in future gene-environment interaction studies on human complex traits.

## Methods

### Estimation of different parameters

Following the random effect model setup in Eqs. (1), (2), and (3), we propose a joint modeling test as in Eq. (4), which requires the estimation of ${h}_{I}^{2}$, ${\rho }_{IG}$, and $\boldsymbol{\Sigma }$. PIGEON [7] uses summary statistics and partial LD information to estimate these three components. LDER-GE [12] uses summary statistics and full LD information to estimate ${h}_{I}^{2}$. Here we propose to use summary statistics and full LD information to estimate ${\rho }_{IG}$ (Eq. (6)) and delete-block wise jackknife to estimate $\boldsymbol{\Sigma }$. The weight used for iterative reweighted least square estimation (Eq. (6)) takes the form of

12 $${w}_{j}=min\left({D}_{jj},1\right)/{T}_{jj},$$

13 $${T}_{jj}=\left({{D}_{jj}N}_{G}{h}_{g}^{2}/M+1\right)\left({D}_{jj}{N}_{I}{h}_{I}^{2}/M+1+2\left({h}_{I}^{2}+{\sigma }_{1}^{2}\right)\right)+{\left(\sqrt{{N}_{G}{N}_{I}}{\rho }_{IG}{D}_{j,j}/M+2{N}_{S}\left({\rho }_{IG}+{\rho }_{01}\right)/\sqrt{{N}_{G}{N}_{I}}\right)}^{2},$$

where ${T}_{jj}$ is proportional to the variance of the transformed Z-score product (Supplementary Note 2) and serves as the optimal regression weight [32], and we further shrink by a factor $min\left({D}_{jj},1\right)$ as ref [12, 17] to balance the noise and information of transformed Z-score product with large eigenvalues. We first estimate ${h}_{g}^{2}$ [17] and ${h}_{I}^{2}, {\sigma }_{1}^{2}$ [12] and plug them into ${w}_{j}$ for the iterative reweighted least square estimation of ${\rho }_{IG}$.

### UKBB data for simulation and real data analysis

We used the UK Biobank [13] as the real data source for this study with application number 29900 for simulations and genomic partitioning. We used UKBB dataset with application number 32285 for real data analysis including GWAS, GWIS, and PRS-by-E analysis. Detailed procedure of data access, ethical approval, quality control procedures, phenotype definitions, and PRS definitions can be found in Supplementary Note 2.

### Reference panel construction

We used the same reference genetic panel for simulation studies and real data analysis as ref [12]. Basically, for simulation studies, we took *M* = 396,330 common variants UKBB genotype imputed variants intersected with the 1000 Genomes project [33] and hapmap3 project [34], on *N* = 276,050 European ancestry subjects. On the same genotype panel (*M* = 396,330, *N* = 276,050), we partitioned the whole genome into 1009 genomic blocks roughly independent from each other to speed up computation [12]. For UKBB real data analysis, we used the in-sample reference panel with *N* = 307,259 European ancestry subjects and *M* = 966,766 variants from UKBB imputed genotype panel and UKBB array genotype panel, intersected with hapmap3 project [34]. Further details of quality control reference panel construction can be found in ref [12] and Supplementary Note 2.

### Simulations

The data generation process followed Eqs. 14 and 15. To examine the effect of joint modeling, we set the narrow-sense heritability ${h}_{g}^{2}\in (\text{0.1,0.2,0.3})$, G × E interaction proportion ${h}_{I}^{2}\in (\text{0.01,0.02,0.03})$, and G × E genetic correlation ${r}_{IG}\in (0, 0.1, 0.2, 0.3, 0.4, 0.5)$. We generated the environment variable E from standard normal distribution. The parameters estimation was conducted using PIGEON and BV-LDER-GE separately to compare the statistical efficiency.

14 $${Y}_{i}={\sum }_{j=1}^{M}{G}_{ji}{\beta }_{j}+{\sum }_{j=1}^{M}{S}_{ji}{\gamma }_{j}+{\epsilon }_{0i},$$

15 $$\left[\begin{array}{c}{\beta }_{j} \\ {\gamma }_{j}\end{array}\right]{\sim }^{iid} N \left( \left[ \begin{array}{c}0\\ 0\end{array} \right], \left[ \begin{array}{cc}{h}_{g}^{2}/M& \frac{{\rho }_{IG}}{M}\\ \frac{{\rho }_{IG}}{M}& {h}_{I}^{2}/M\end{array} \right]\right).$$

For each simulation, we randomly chose 20,000 subjects and M0 = 19,816 (5%) causal GE and additive effect variants for data generation, association analysis (linear regression on *M* = 396,330 variants using PLINK2 [35]) and G × E interaction parameter estimation analysis. We repeated 500 times for each simulation scenario.

To compare the performance of correcting the confounding effect brought my heritable environmental covariates, we followed Eqs. (7) and (8) to generate variables. We fix the narrow-sense heritability of E and Y at ${h}_{g}^{2}={h}_{e}^{2}=0.3$, and their genetic correlation ${r}_{GE}=0.5$. We set G × E interaction proportion ${h}_{I}^{2}\in (0.03, 0.05)$, and ${r}_{GE}=0.5, {r}_{IE}\in (\text{0.3,0.5})$ to explore different parameter scenarios.

### Variant-level association analysis of UKBB

We analyzed 14 continuous phenotypes and eight binary phenotypes, paired with seven environmental covariates, resulting in a total of 151 (22*7 − 3) E-Y pairs with three sex-specific phenotypes. GWIS analysis was conducted through “–glm interaction –variance-standardize” command of PLINK2 [35] pre-adjusted for age, sex, 40 genetic PCs, and the specific environmental covariate of interest. The command automatically output the GWAS result for additive genetic effect on the phenotype. To adjust potential confounding effect when the environmental covariate is heritable, we obtained the summary statistics on the environmental covariate using “–glm allow-no-covars” command of PLINK2 [35]. Specifically, the three heritable environmental covariates are BMI, alcohol intake, and packed years of smoking.

### PRS fitting methods and PRS-by-E effect linear regression

We use GWAS summary statistics from European populations that are publicly accessible for 13 traits and predicted PRS for UK Biobank samples (Additional file 2: Table S8). There is no overlap between the samples in the UK Biobank and the individuals in the GWAS summary statistics.

For SBP and DBP, we split UKBB cohort based on the genotype batch, using the first 35 batches as the training set and the last 71 batches as the testing set [4]. We used Plink2 to run the GWAS analysis in the training set and fit the PRS to the testing set for the subsequent linear regression analysis.

We then fit the linear regression model via $Y\sim PRS\_Y+E+PRS\_Y*E+AGE+SEX$ for the 15 phenotypes and 7 environmental covariates (excluding sex-breast cancer pair) in unrelated UKBB subjects with European ancestry (total *N* = 307,259). The all variables including PRS are standardized before running linear regression.

## Supplementary Information

Additional file 1: The theoretical and statistical derivation details of the model BV-LDER-GE.

Additional file 2: UKBB genotype data quality control details, phenotypic definition details, and discussion on reference panel. Supplementary Tables 7–9.

Additional file 3: Supplementary Tables 1–6.

## Data Availability

The source code of BV-LDER-GE is available on Github and Zenodo under the MIT License. The public available dataset used in this study is UK-BioBank Dataset.
