# Supplementary material for: Incorporating additive genetic effects and linkage disequilibrium information to discover gene-environment interactions using BV-LDER-GE
Source: Genome Biol. 2025 Oct 3;26:332. doi: 10.1186/s13059-025-03815-z (PMC12492645; doi:10.1186/s13059-025-03815-z)
Supplement: Supplementary file 1 — Additional file 1: The theoretical and statistical derivation details of the model BV-LDER-GE. [file 13059_2025_3815_MOESM1_ESM.pdf]

Supplementary note one of Bivariate-LDER-GE

Zihan Dong, Wei Jaing, Andrew DeWan, Hongyu Zhao

1 Main derivation

We set up the model

$$Y_i = \sum_{j=1}^{M} G_{ji}\beta_j + \sum_{j=1}^{M} S_{ji}\gamma_j + \epsilon_{1i}E_i + \epsilon_{0i} \tag{1}$$

Where Y_i is the phenotype for subject i already adjusted for fixed effect including for the exposure covariate. E_i is the standardized exposure covariate for subject i. Suppose there are M variant. G_{ji} is the jth variant for subject i. $S_{ji} = G_{ji} * E_i$ is the GE interaction product term for variant j of subject i. ϵ_{1i} is the non-genetic residual that has exposure interaction effect. ϵ_{0i} is the residual independent from all other parts.β_j is the true additive effect for variant j. γ_j is the true interaction effect for variant j.

In matrix form, it's written as

$$\boldsymbol{Y} = \boldsymbol{G\beta} + \boldsymbol{S\gamma} + \boldsymbol{\epsilon_1 E} + \boldsymbol{\epsilon_0} \tag{2}$$

We model β_j and γ_j using random effect model:

$$\begin{pmatrix}\beta_j \\ \gamma_j\end{pmatrix} \sim N\left[\begin{pmatrix}0 \\ 0\end{pmatrix}, \begin{pmatrix} h_g^2/M & \rho_{gI}/M \\ \rho_{gI}/M & h_I^2/M \end{pmatrix}\right] \tag{3}$$

Where h_g^2 is the narrow-sense heritability and h_I^2 is the GE interaction contribution proportion that we are interested at. ρ_{gI} models the genome-level covariance between the additive effect and GE interaction effect.
We model ϵ_0 and ϵ_1 using random effect model:

$$\begin{pmatrix}\epsilon_{0_i} \\ \epsilon_{1_i}\end{pmatrix} \sim N\left[\begin{pmatrix}0 \\ 0\end{pmatrix}, \begin{pmatrix} \sigma_0^2 & \rho_{0,1} \\ \rho_{0,1} & \sigma_1^2 \end{pmatrix}\right] \tag{4}$$

We assume that the genetic part and residual part are independent.

Suppose all variants and the exposure variable are standardized.
Then the main genetic effect estimate for variant j is:

$$\begin{aligned}\hat{\beta_j} &= (\boldsymbol{G_{j.}^{(G)T}} \boldsymbol{G_{j.}^{(G)}})^{-1} \boldsymbol{G_{j.}^{(G)T}} \boldsymbol{Y} \\ &= \boldsymbol{G_{j.}^{(G)T}} \boldsymbol{Y^{(G)}}/N_G\end{aligned} \tag{5}$$

Where the superscript (G) represents the sample of the main genetic association with N_G being its sample size. The

first equality holds because after standardization $\text{var}(G_{j.}) = 1$
And the Z score of the main genetic effect estimate for variant j is:

$$Z_{jG} = \boldsymbol{G_{j.}^{(G)T} Y^{(G)}} / \sqrt{N_G} \tag{6}$$

The interaction effect estimate for variant k is:

$$\begin{aligned} \hat{\gamma}_j &= \boldsymbol{(S_{k.}^{(I)T} S_{k.}^{(I)})}^{-1} \boldsymbol{S_{k.}^{(I)T} Y^{(I)}} \\ &= \boldsymbol{S_{k.}^{(I)T} Y^{(I)}} / N_I \end{aligned} \tag{7}$$

Where the superscript (I) represents the sample of the GE interaction association with N_I being its sample size, which maybe different from (G). Denote the overlap sample size as N_S. The first equality holds because after standardization $\text{var}(S_{k.}) = 1$.
And the Z score of the interaction effect estimate for variant k is:

$$Z_{kI} = \boldsymbol{S_{k.}^{(I)T} Y^{(I)}} / \sqrt{N_I} \tag{8}$$

Then for two variant j and k, the expected corss-variant Z score product is:

$$\begin{aligned} \mathbb{E}(Z_{jG} Z_{kI}) &= \mathbb{E}(\mathbb{E}(Z_{jG} Z_{kI} | \boldsymbol{\beta}, \boldsymbol{\gamma}, \boldsymbol{\epsilon_0}, \boldsymbol{\epsilon_1})) \\ &= \frac{1}{\sqrt{N_G N_I}} \mathbb{E}(\mathbb{E}(\boldsymbol{G_{j.}^{(G)T} Y^{(G)} S_{k.}^{(I)T} Y^{(I)}} | \boldsymbol{\beta}, \gamma, \boldsymbol{\epsilon_0}, \boldsymbol{\epsilon_1})) \\ &= \frac{1}{\sqrt{N_G N_I}} \mathbb{E}(\boldsymbol{G_{j.}^{(G)T} (G^{(G)} \beta + S^{(G)}} \gamma + \boldsymbol{\epsilon_1^{(G)} E^{(G)} + \epsilon_0^{(G)}})) \\ & (\boldsymbol{S_{k.}^{(I)T} (G^{(I)} \beta + S^{(I)}} \gamma + \boldsymbol{\epsilon_1^{(I)} E^{(I)} + \epsilon_0^{(I)}})) \\ &= \frac{1}{\sqrt{N_G N_I}} \mathbb{E}(\boldsymbol{G_{j.}^{(G)T} G^{(G)} \beta S_{k.}^{(I)T} S^{(I)}} \gamma) \\ &+ \frac{1}{\sqrt{N_G N_I}} \mathbb{E}(\boldsymbol{G_{j.}^{(G)T} S^{(G)} \beta S_{k.}^{(I)T} G^{(I)}} \gamma) \\ &+ \frac{1}{\sqrt{N_G N_I}} \mathbb{E}(\boldsymbol{G_{j.}^{(G)T} \epsilon_1^{(G)} E^{(G)} S_{k.}^{(I)T} \epsilon_0^{(I)}}) \\ &+ \frac{1}{\sqrt{N_G N_I}} \mathbb{E}(\boldsymbol{G_{j.}^{(G)T} \epsilon_0^{(G)} S_{k.}^{(I)T} \epsilon_1^{(I)} E^{(I)}}) \\ &= \frac{1}{\sqrt{N_G N_I}} (\frac{\rho_{g,I}}{M}((N_G N_I + N_S) \ell_{j,k} + N_S M r_{j,k}) + \frac{\rho_{g,I}}{M} N_S (2\ell_{j,k} + M r_{j,k}) + 2\rho_{0,1} N_S r_{j,k}) \\ &\approx \frac{\sqrt{N_G N_I} \rho_{g,I}}{M} \ell_{j,k} + \frac{2N_S}{\sqrt{N_G N_I}} (\rho_{0,1} + \rho_{g,I}) r_{j,k} \end{aligned} \tag{9}$$

where r_{jk} is the true correlation between the SNP j and k, and $\ell_{jk} = \sum_{t=1}^{M} r_{jt} r_{kt}$ is the off diagonal element of the LD score matrix. The details of the forth equality are in supplementary.
In matrix form, the above quantity can be written as

$$\mathbb{COV}(\boldsymbol{Z_G}, \boldsymbol{Z_I}) = \frac{\sqrt{N_G N_I} \rho_{g,I}}{M} \boldsymbol{L} + \frac{2N_S}{\sqrt{N_G N_I}} (\rho_{0,1} + \rho_{g,I}) \boldsymbol{R} \tag{10}$$

where $\boldsymbol{L} = \boldsymbol{R^T R}$ is the LD score matrix.

2 Details

This section provides the derivation details.
For equation (9) equality 4 there are 4 parts omitting constant term $\frac{1}{\sqrt{N_G N_I}}$ for convenience:

$$\begin{aligned}
&\mathbb{E}(\boldsymbol{G_{j.}^{(G)T}}\boldsymbol{G^{(G)}}\boldsymbol{\beta}\boldsymbol{S_{k.}^{(I)T}}\boldsymbol{S^{(I)}}\gamma) + \mathbb{E}(\boldsymbol{G_{j.}^{(G)T}}\boldsymbol{S^{(G)}}\boldsymbol{\beta}\boldsymbol{S_{k.}^{(I)T}}\boldsymbol{G^{(I)}}\gamma) \\
&+ \mathbb{E}(\boldsymbol{G_{j.}^{(G)T}}\boldsymbol{\epsilon_1^{(G)}}\boldsymbol{E^{(G)}}\boldsymbol{S_{k.}^{(I)T}}\boldsymbol{\epsilon_0^{(I)}}) + \mathbb{E}(\boldsymbol{G_{j.}^{(G)T}}\boldsymbol{\epsilon_0^{(G)}}\boldsymbol{S_{k.}^{(I)T}}\boldsymbol{\epsilon_1^{(I)}}\boldsymbol{E^{(I)}}) \\
&= (\frac{\rho_{g,I}}{M}(N_G N_I \ell_{j,k} + N_S M r_{j,k}) + \frac{\rho_{g,I}}{M} N_S(2\ell_{j,k} + M r_{j,k}) + 2\rho_{0,1} N_S r_{j,k})
\end{aligned} \tag{11}$$

The first part:

$$\begin{aligned}
&\mathbb{E}(\boldsymbol{G_{j.}^{(G)T}}\boldsymbol{G^{(G)}}\boldsymbol{\beta}\boldsymbol{S_{k.}^{(I)T}}\boldsymbol{S^{(I)}}\gamma) \\
&= \frac{\rho_{g,I}}{M}\mathbb{E}(\sum_{t=1}^{M} \boldsymbol{S_{j,.}^T}\boldsymbol{S_{t,.}}\boldsymbol{G_{k,.}^T}\boldsymbol{G_{t,.}}) \\
&= \frac{\rho_{g,I}}{M}\mathbb{E}(\sum_{t=1}^{M}(\sum_{a=1}^{N_I} E_a^2 G_{j,a}G_{t,a})(\sum_{b=1}^{N_G} G_{k,b}G_{t,b})) \\
&= \frac{\rho_{g,I}}{M}\sum_{t=1}^{M}\sum_{a=1}^{N_I}\sum_{b=1}^{N_G}\mathbb{E}(G_{j,a}G_{t,a}G_{k,b}G_{t,b}) \\
&= \frac{\rho_{g,I}}{M}((N_G N_I + N_S)\ell_{j,k} + N_S M r_{j,k})
\end{aligned} \tag{12}$$

The forth equality utilizes Isserlis' Theorem:

$$\begin{aligned}
&\mathbb{E}(G_{j,a}G_{t,a}G_{k,b}G_{t,b}) = \\
&\mathbb{E}(G_{j,a}G_{t,a})\mathbb{E}(G_{k,b}G_{t,b}) + \mathbb{E}(G_{j,a}G_{k,b})\mathbb{E}(G_{t,a}G_{t,b}) + \mathbb{E}(G_{j,a}G_{t,b})\mathbb{E}(G_{t,a}G_{k,b}) \\
&= r_{j,t}r_{k,t} + \boldsymbol{I}(a=b)r_{j,k} + \boldsymbol{I}(a=b)r_{j,t}r_{k,t}
\end{aligned} \tag{13}$$

Where $\boldsymbol{I}$ is the indicator operation.
The second part:

$$\begin{aligned}
&\mathbb{E}(\boldsymbol{G_{j.}^{(G)T}}\boldsymbol{S^{(G)}}\boldsymbol{\beta}\boldsymbol{S_{k.}^{(I)T}}\boldsymbol{G^{(I)}}\gamma) \\
&= \frac{\rho_{g,I}}{M}\mathbb{E}(\sum_{t=1}^{M} \boldsymbol{G_{j,.}^T}\boldsymbol{S_{t,.}}\boldsymbol{S_{k,.}^T}\boldsymbol{G_{t,.}}) \\
&= \frac{\rho_{g,I}}{M}\mathbb{E}(\sum_{t=1}^{M}(\sum_{a=1}^{N_I} E_a G_{j,a}S_{t,a})(\sum_{b=1}^{N_G} E_b G_{k,b}S_{t,b}) \\
&= \frac{\rho_{g,I}}{M}\sum_{t=1}^{M}\sum_{a=1}^{N_I}\sum_{b=1}^{N_G}\mathbb{E}(E_a E_b G_{j,a}G_{t,a}G_{k,b}G_{t,b}) \\
&= \frac{\rho_{g,I}}{M}\sum_{t=1}^{M}\sum_{a=1}^{N_I}\sum_{b=1}^{N_G}\boldsymbol{I}(a=b)\mathbb{E}(G_{j,a}G_{t,a}G_{k,b}G_{t,b}) \\
&= \frac{\rho_{g,I}}{M}\sum_{t=1}^{M}(2\ell_{j,k} + M r_{j,k})
\end{aligned} \tag{14}$$

The derivation of the fifth equality can be found in equation 17 and 18.
The third part:

$$\begin{aligned}
&\mathbb{E}(\boldsymbol{G_{j.}^{(G)T}} \boldsymbol{\epsilon_1^{(G)}} \boldsymbol{E^{(G)}} \boldsymbol{S_{k.}^{(I)T}} \boldsymbol{\epsilon_0^{(I)}}))\\
&= \rho_{0,1}\mathbb{E}(\sum_{a=1}^{N_I} G_{j,a}E_a \sum_{b=1}^{N_G} E_b G_{k,b})\\
&= \rho_{0,1}\mathbb{E}(\sum_{a=1}^{N_I}\sum_{b=1}^{N_G} \boldsymbol{I}(a=b)E_aE_bG_{j,a}G_{k,b})\\
&= \rho_{0,1}N_S r_{j,k}
\end{aligned} \tag{15}$$

The forth part:

$$\begin{aligned}
&\mathbb{E}(\boldsymbol{G_{j.}^{(G)T}} \boldsymbol{\epsilon_0^{(G)}} \boldsymbol{S_{k.}^{(I)T}} \boldsymbol{\epsilon_1^{(I)}} \boldsymbol{E^{(I)}})\\
&= \rho_{0,1}\mathbb{E}(\sum_{a=1}^{N_I} G_{j,a} \sum_{b=1}^{N_G} E_b^2 G_{k,b})\\
&= \rho_{0,1}\mathbb{E}(\sum_{a=1}^{N_I}\sum_{b=1}^{N_G} \boldsymbol{I}(a=b)E_b^2G_{j,a}G_{k,b})\\
&= \rho_{0,1}N_S r_{j,k}
\end{aligned} \tag{16}$$

To show the sixth equality in equation 14, we model three standardized variants G_{jG}, G_{ki}, G_{ti} for one subject i as MVN distribution.

$$\begin{pmatrix} G_{jG} \\ G_{ki} \\ G_{ti} \end{pmatrix} \sim N\left[\begin{pmatrix} 0 \\ 0 \\ 0 \end{pmatrix}, \begin{pmatrix} 1 & r_{jk} & r_{jt} \\ r_{jk} & 1 & r_{kt} \\ r_{jt} & r_{kt} & 1 \end{pmatrix}\right] \tag{17}$$

Then, by low of total expectation,

$$\begin{aligned}
&\mathbb{E}(G_{ji}G_{ki}G_{ti}^2)\\
&= \mathbb{E}(\boldsymbol{E}(G_{ji}G_{ki}G_{ti}^2|G_{ti})\\
&= 2r_{jt}r_{kt} + r_{jk}
\end{aligned} \tag{18}$$

3 Heritable exposure covariate E

When the exposure E is heritable, where α is the true polygenic effect modeled as a random effect, and ϵ_e is the residual to make E standardized.

$$\boldsymbol{E} = \boldsymbol{G\alpha} + \boldsymbol{\epsilon_e} \tag{19}$$

$$\begin{pmatrix} \beta_j \\ \gamma_j \\ \alpha_j \end{pmatrix} \sim N\left[\begin{pmatrix} 0 \\ 0 \\ 0 \end{pmatrix}, \begin{pmatrix} h_g^2/M & \rho_{gI}/M & \rho_{ge}/M \\ \rho_{gI}/M & h_I^2/M & \rho_{eI}/M \\ \rho_{ge}/M & \rho_{eI}/M & h_e^2/M \end{pmatrix}\right] \tag{20}$$

$$\tag{21}$$

Miao et al in [1] models as follows:

$$\begin{aligned} \boldsymbol{Y} &= \boldsymbol{G\beta} + \boldsymbol{S\gamma} + \boldsymbol{\epsilon_1 E} + \boldsymbol{\epsilon_0} \\ \boldsymbol{E} &= \boldsymbol{G\alpha} + \boldsymbol{\epsilon_e} \\ \boldsymbol{Y_2} &= \boldsymbol{Z\theta} + \boldsymbol{\epsilon_2} \end{aligned} \tag{22}$$

Where $\boldsymbol{Z}$ is the genotype panel for $\boldsymbol{Y_2}$ (same or different from the sample of $\boldsymbol{Y}$), and θ is its effect sizes. We denote $\rho_{G2I} = Cov(\theta_j, \gamma_j)$ and $\rho_{G2E} = Cov(\theta_j, \alpha_j)$.Miao et al derived the bivariate product of interaction analysis on Y and main genetic effect analysis on Y_2 for the same variant, and we extend this to two possibly different variant j and k based on their previous work:

$$\begin{aligned} \mathbb{E}(Z_{jG_2} Z_{kI} | \boldsymbol{G}, \boldsymbol{Z}, \boldsymbol{E}) &= \frac{1}{\sqrt{N_{G2} N_I}} \mathbb{E}(\boldsymbol{Z_{j.}^T Y_2 S_{k.}^T Y}) \\ &= \frac{1}{\sqrt{N_{G2} N_I}} (T1 + T2 - T3) \end{aligned} \tag{23}$$

Where

$$\begin{aligned} T1 &= \frac{\rho_{\theta I}}{M} \boldsymbol{Z_{j.}^T Z I_M S^T S_{k.}} \\ T2 &= (diag(\boldsymbol{G\alpha})\boldsymbol{G})_k^T (diag(\boldsymbol{G\alpha})\boldsymbol{G})\gamma \theta^T \boldsymbol{Z^T Z_j} \\ T3 &= (diag(\boldsymbol{G\alpha})\boldsymbol{G})_k^T \mathbb{E}[(diag(\boldsymbol{G\alpha})\boldsymbol{G})\gamma] \theta^T \boldsymbol{Z^T Z_j} \end{aligned} \tag{24}$$

From equation (9) we derive that

$$\begin{aligned} \mathbb{E}(T1) &= \mathbb{E}(\frac{\rho_{\theta I}}{M} \boldsymbol{Z_{j.}^T Z I_M S^T S_{k.}}) \\ &= (1 - h_e^2)(N_I N_{G2} \ell_{jk} \frac{\rho_{G2I}}{M} + N_S \rho_{G2I} r_{jk}) \end{aligned} \tag{25}$$

Following Miao et al's derivation, for T2 we generalize to two possibly different variants j and k:

$$\begin{aligned} \mathbb{E}(T2) &= \mathbb{E}[(diag(\boldsymbol{G\alpha})\boldsymbol{G})_k^T (diag(\boldsymbol{G\alpha})\boldsymbol{G})\gamma \theta^T \boldsymbol{Z^T Z_j}] \\ &= \sum_{i=1}^{N_I} \sum_{q=1}^{N_{G2}} \sum_{a=1}^{M} \sum_{b=1}^{M} \sum_{c=1}^{M} \sum_{d=1}^{M} \mathbb{E}[G_{ik} G_{ia} \alpha_a G_{ib} \gamma_b G_{ic} \gamma_c Z_{qj} Z_{qd} \theta_d] \\ &= \sum_{i=1}^{N_I} \sum_{q=1}^{N_{G2}} \sum_{a=1}^{M} \sum_{b=1}^{M} \sum_{c=1}^{M} \sum_{d=1}^{M} \mathbb{E}[G_{ik} G_{ia} G_{ib} G_{ic} Z_{qj} Z_{qd}] \mathbb{E}[\alpha_a \gamma_b \gamma_c \theta_d] \\ &= N_I N_{G2} (h_e^2 \rho_{G2,I} + 2M \mathbb{E}(\alpha_a \gamma_a) \mathbb{E}(\alpha_a \theta_a)) \ell_{jk} + N_S (h_e^2 \rho_{G2I} + 2M \mathbb{E}(\alpha_a \gamma_a) \mathbb{E}(\alpha_a \theta_a)) r_{jk} \end{aligned} \tag{26}$$

Where the last equality is justified using Isserlis' theorem. For the third term, we did similar derivation:

$$\begin{aligned} \mathbb{E}(T3) &= \mathbb{E}[(diag(\boldsymbol{G\alpha})\boldsymbol{G})_k^T \mathbb{E}[(diag(\boldsymbol{G\alpha})\boldsymbol{G})\gamma] \theta^T \boldsymbol{Z^T Z_j}] \\ &= N_I N_{G2} M \mathbb{E}(\alpha_a \gamma_a) \mathbb{E}(\alpha_a \theta_a) \ell_{jk} + N_s \mathbb{E}(\alpha_a \gamma_a) \mathbb{E}(\alpha_a \theta_a) r_{jk} \end{aligned} \tag{27}$$

Merging three terms T1, T2 and T3, we get

$$\begin{aligned} \mathbb{E}(Z_{jG_2} Z_{kI}) &= \mathbb{E}(\mathbb{E}(Z_{jG_2} Z_{kI} | \boldsymbol{G}, \boldsymbol{Z}, \boldsymbol{E})) \\ &= \mathbb{E}(T1) + \mathbb{E}(T2) + \mathbb{E}(T3) \\ &= \frac{\sqrt{N_I N_{G2}} (\rho_{G2I} + \rho_{G2E} \rho_{eI})}{M} \ell_{jk} + c_1 r_{jk} \end{aligned} \tag{28}$$

Where c1 is the intercept which incorporates potential confounding effects. When Y2 is E, we get

$$\mathbb{E}(Z_{jE}Z_{kI}) = \frac{\sqrt{N_I N_E}(\rho_{eI} + \rho_{eI}h_e^2)}{M}\ell_{jk} + c_1 r_{jk} \tag{29}$$

When Y2 is Y, we get

$$\mathbb{E}(Z_{jG}Z_{kI}) = \frac{\sqrt{N_I N_G}(\rho_{gI} + \rho_{ge}\rho_{eI})}{M}\ell_{jk} + c_2 r_{jk} \tag{30}$$

We can utilize equation (29) to estimate ρ_{eI} and equation (30) to estimate ρ_{gI}.

$$\begin{aligned}
\mathbb{E}(Z_{jI}Z_{kI}) &= \mathbb{E}(\mathbb{E}(Z_{jI}Z_{kI}|\boldsymbol{\beta}, \boldsymbol{\gamma}, \boldsymbol{\alpha}, \boldsymbol{\epsilon_0}, \boldsymbol{\epsilon_1}, \boldsymbol{\epsilon_e})) \\
&= \mathbb{E}(\mathbb{E}(\boldsymbol{S_{j.}^T Y S_{k.}^T Y}/n|\boldsymbol{\beta}, \boldsymbol{\gamma}, \boldsymbol{\alpha}, \boldsymbol{\epsilon_0}, \boldsymbol{\epsilon_1}, \boldsymbol{\epsilon_e})) \\
&= \mathbb{E}(\boldsymbol{S_{j.}^T(G\beta + S\gamma + \epsilon_1 E + \epsilon_0)S_{k.}^T(G\beta + S\gamma + \epsilon_1 E + \epsilon_0)})/n \\
&= \mathbb{E}(\boldsymbol{S_{j.}^T(G\beta)S_{k.}^T(G\beta)})/n + \mathbb{E}(\boldsymbol{S_{j.}^T(S\gamma)S_{k.}^T(S\gamma)})/n \\
&+ \mathbb{E}(\boldsymbol{S_{j.}^T(\epsilon_1 E)S_{k.}^T(\epsilon_1 E)})/n + \mathbb{E}(\boldsymbol{S_{j.}^T(\epsilon_0)S_{k.}^T(\epsilon_0)})/n \\
&= \mathbb{E}([diag(\boldsymbol{G\alpha} + \boldsymbol{\epsilon_e})\boldsymbol{G}]_{j.}^T(\boldsymbol{G\beta})[diag(\boldsymbol{G\alpha} + \boldsymbol{\epsilon_e})\boldsymbol{G}]_{k.}^T(\boldsymbol{G\beta}))/n \\
&+ \mathbb{E}([diag(\boldsymbol{G\alpha} + \boldsymbol{\epsilon_e})\boldsymbol{G}]_{j.}^T diag(\boldsymbol{G\alpha} + \boldsymbol{\epsilon_e})\boldsymbol{G\gamma}[diag(\boldsymbol{G\alpha} + \boldsymbol{\epsilon_e})\boldsymbol{G}]_{k.}^T diag(\boldsymbol{G\alpha} + \boldsymbol{\epsilon_e})\boldsymbol{G\gamma}/n \\
&+ \mathbb{E}([diag(\boldsymbol{G\alpha} + \boldsymbol{\epsilon_e})\boldsymbol{G}]_{j.}^T(\boldsymbol{\epsilon_1 E})[diag(\boldsymbol{G\alpha} + \boldsymbol{\epsilon_e})\boldsymbol{G}]_{k.}^T(\boldsymbol{\epsilon_1 E}))/n \\
&+ \mathbb{E}([diag(\boldsymbol{G\alpha} + \boldsymbol{\epsilon_e})\boldsymbol{G}]_{j.}^T(\boldsymbol{\epsilon_0})[diag(\boldsymbol{G\alpha} + \boldsymbol{\epsilon_e})\boldsymbol{G}]_{k.}^T(\boldsymbol{\epsilon_0}))/n \\
&= \mathbb{E}([diag(\boldsymbol{G\alpha})\boldsymbol{G}]_{j.}^T(\boldsymbol{G\beta})[diag(\boldsymbol{G\alpha})\boldsymbol{G}]_{k.}^T(\boldsymbol{G\beta}))/n + \mathbb{E}([diag(\boldsymbol{\epsilon_e})\boldsymbol{G}]_{j.}^T(\boldsymbol{G\beta})[diag(\boldsymbol{\epsilon_e})\boldsymbol{G}]_{k.}^T(\boldsymbol{G\beta}))/n \\
&+ \mathbb{E}([diag(\boldsymbol{G\alpha})\boldsymbol{G}]_{j.}^T diag(\boldsymbol{G\alpha})\boldsymbol{G\gamma}[diag(\boldsymbol{G\alpha})\boldsymbol{G}]_{k.}^T diag(\boldsymbol{G\alpha})\boldsymbol{G\gamma}/n \\
&+ \mathbb{E}([diag(\boldsymbol{\epsilon_e})\boldsymbol{G}]_{j.}^T diag(\boldsymbol{\epsilon_e})\boldsymbol{G\gamma}[diag(\boldsymbol{\epsilon_e})\boldsymbol{G}]_{k.}^T diag(\boldsymbol{\epsilon_e})\boldsymbol{G\gamma}/n \\
&+ \mathbb{E}([diag(\boldsymbol{G\alpha})\boldsymbol{G}]_{j.}^T(\boldsymbol{\epsilon_1 E})[diag(\boldsymbol{G\alpha})\boldsymbol{G}]_{k.}^T(\boldsymbol{\epsilon_1 E}))/n + \mathbb{E}([diag(\boldsymbol{\epsilon_e})\boldsymbol{G}]_{j.}^T(\boldsymbol{\epsilon_1 E})[diag(\boldsymbol{\epsilon_e})\boldsymbol{G}]_{k.}^T(\boldsymbol{\epsilon_1 E}))/n \\
&+ \mathbb{E}([diag(\boldsymbol{G\alpha})\boldsymbol{G}]_{j.}^T(\boldsymbol{\epsilon_0})[diag(\boldsymbol{G\alpha})\boldsymbol{G}]_{k.}^T(\boldsymbol{\epsilon_0}))/n + \mathbb{E}([diag(\boldsymbol{\epsilon_e})\boldsymbol{G}]_{j.}^T(\boldsymbol{\epsilon_0})[diag(\boldsymbol{\epsilon_e})\boldsymbol{G}]_{k.}^T(\boldsymbol{\epsilon_0}))/n
\end{aligned} \tag{31}$$

Recognizing that ϵ_e can be regarded the unstandardized non-heritable exposure covariate. Standardizing ϵ_e by multiplying $\frac{1}{\sqrt{1-h_e^2}}$ and utilizing the conclusion from the previous LDER-GE study [2], we have that

$$\begin{aligned}
&\mathbb{E}([diag(\boldsymbol{\epsilon_e})\boldsymbol{G}]_{j.}^T(\boldsymbol{G\beta})[diag(\boldsymbol{\epsilon_e})\boldsymbol{G}]_{k.}^T(\boldsymbol{G\beta}))/n + \mathbb{E}([diag(\boldsymbol{\epsilon_e})\boldsymbol{G}]_{j.}^T diag(\boldsymbol{\epsilon_e})\boldsymbol{G\gamma}[diag(\boldsymbol{\epsilon_e})\boldsymbol{G}]_{k.}^T diag(\boldsymbol{\epsilon_e})\boldsymbol{G\gamma}/n \\
&+ \mathbb{E}([diag(\boldsymbol{\epsilon_e})\boldsymbol{G}]_{j.}^T(\boldsymbol{\epsilon_1 E})[diag(\boldsymbol{\epsilon_e})\boldsymbol{G}]_{k.}^T(\boldsymbol{\epsilon_1 E}))/n + \mathbb{E}([diag(\boldsymbol{\epsilon_e})\boldsymbol{G}]_{j.}^T(\boldsymbol{\epsilon_0})[diag(\boldsymbol{\epsilon_e})\boldsymbol{G}]_{k.}^T(\boldsymbol{\epsilon_0}))/n \\
&\approx (1-h_e^2)h_g^2 M r_{jk}/M + (1-h_e^2)^2 h_I^2/M * ((n^2 + 2nK(E) - 2n)\ell_{jk} + nMK(E)r_{jk})/n \\
&+ (1-h_e^2)\sigma_1^2 K(E) r_{jk} + (1-h_e^2)\sigma_0^2 r_{jk}
\end{aligned} \tag{32}$$

Following the derivations for (26) and (27) and the Isserlis' theorem, we get

$$\begin{aligned}
&\mathbb{E}([diag(\boldsymbol{G\alpha})\boldsymbol{G}]_{j.}^T(\boldsymbol{G\beta})[diag(\boldsymbol{G\alpha})\boldsymbol{G}]_{k.}^T(\boldsymbol{G\beta}))/n + \mathbb{E}([diag(\boldsymbol{G\alpha})\boldsymbol{G}]_{j.}^T diag(\boldsymbol{G\alpha})\boldsymbol{G\gamma}[diag(\boldsymbol{G\alpha})\boldsymbol{G}]_{k.}^T diag(\boldsymbol{G\alpha})\boldsymbol{G\gamma}/n \\
&+ \mathbb{E}([diag(\boldsymbol{G\alpha})\boldsymbol{G}]_{j.}^T(\boldsymbol{\epsilon_1 E})[diag(\boldsymbol{G\alpha})\boldsymbol{G}]_{k.}^T(\boldsymbol{\epsilon_1 E}))/n + \mathbb{E}([diag(\boldsymbol{G\alpha})\boldsymbol{G}]_{j.}^T(\boldsymbol{\epsilon_0})[diag(\boldsymbol{G\alpha})\boldsymbol{G}]_{k.}^T(\boldsymbol{\epsilon_0}))/n \\
&\approx (2h_e^2\rho_{eI}^2 + (h_e^2)^2 h_I^2 + 2(1-h_e^2)(h_e^2 h_I^2 + \rho_{eI}^2))\ell_{jk} n/m + (h_e^2 h_g^2 + h_e^2 h_I^2) r_{jk}
\end{aligned} \tag{33}$$

Merging the above two components, we get

$$\mathbb{E}(Z_{jI}Z_{kI}) = \frac{n(h_I^2 + 2\rho_{eI}^2)\ell_{jk}}{M} + c_3 r_{jk} \tag{34}$$

We can use the estimated $\hat{\rho_{eI}}$ from equation (29) and equation (34) to estimate the adjusted h_I^2.

References

[1] J. Miao, G. Song, Y. Wu, J. Hu, Y. Wu, S. Basu, J. S. Andrews, K. Schaumberg, J. M. Fletcher, L. L. Schmitz, *et al.*, "Reimagining gene-environment interaction analysis for human complex traits," *bioRxiv*, pp. 2022–12, 2022.

[2] Z. Dong, W. Jiang, H. Li, A. T. Dewan, and H. Zhao, "Lder-ge estimates phenotypic variance component of gene-environment interactions in human complex traits accurately with ge interaction summary statistics and full ld information," *bioRxiv*, pp. 2023–11, 2023.
