# Supplementary material for: Incorporating additive genetic effects and linkage disequilibrium information to discover gene-environment interactions using BV-LDER-GE
Source: Genome Biol. 2025 Oct 3;26:332. doi: 10.1186/s13059-025-03815-z (PMC12492645; doi:10.1186/s13059-025-03815-z)
Supplement: Supplementary file 2 — Additional file 2: UKBB genotype data quality control details, phenotypic definition details, and discussion on reference panel. Supplementary Tables 7–9. [file 13059_2025_3815_MOESM2_ESM.docx]

Supplementary note two of Bivariate-LDER-GE

**Real data analysis UKBB dataset quality control and phenotype definition**

Participants were enrolled in the UK Biobank, and data accessed under an approved agreement (application ID 32285). Genotypes were assayed on either the UK BiLEVE array or the UK Biobank Axiom array with 733,332 autosomal variants overlapping between the two arrays. Genotype imputation based on Haplotype Reference Consortium and 1000 Genome project panel yielded 93 million variants for each subject. Subject and variant quality control is described in Supplemental Table S7, with subjects limited to those who self-defined and were genetically confirmed as White British (field 22006) and with a call rate >99% variants. We limited the dataset by using the indicator variable (field 22021) which indicates that the subjects were unrelated (at least 3^rd^ degree relatives) and included in the original principal components (PC) calculation. This resulted in a subset of 307,259 unrelated individuals for analysis. Final variant quality control steps for array and hapmap3 imputed SNPs were call rate >99%, Hardy-Weinberg Equilibrium (HWE) p-value > 5x10^-8^, and minor allele frequency (MAF) > 0.05.

Phenotype definition:

SBP: Systolic blood pressure measured by taking two automated (field 4080; N=472,254 subjects) or manual (field 94; N=43,795 subjects) readings. When there were automated and manual readings available, the automated readings were used. The average of the two readings was used as the SBP value. To account for the use of blood pressure lowering medications, we added 15mmHg to the SBP value for all subjects taking one or more blood pressure lower medications^1^. SBP was transformed by rank-based inverse normal transformation in R.

DBP: Diastolic blood pressure measured by taking two automated (field 4080; N=472,254 subjects) or manual (field 94; N=43,795 subjects) readings. When there were automated and manual readings available, the automated readings were used. The average of the two readings was used as the DBP value. To account for the use of blood pressure lowering medications, we added 10mmHg to the DBP value for all subjects taking one or more blood pressure lower medications^1^. SBP was transformed by rank-based inverse normal transformation in R.

ApolipoproteinB: filed 30640.

Glucose: field 30740.

HbA1c: field 30750.

Health_rating: Overall health rating, field 2178. We transformed the categorical scale to integer scale: “Excellent” -> 4; “Good” -> 3; “Fair” -> 2; “Poor” -> 1. “Do not know” and “Prefer not to answer” were excluded.

Height: standing height, field 50.

Neuro_score: Neuroticism score, field 20127.

CAD: Coronary artery disease, ICD-10 code (field 41270, I210-I214, I219, K401-K404, K411-K414, K451-K455, K491, K492, K498, K499, K502, K751-K754, K758, K759).

T2D: type II diabetes, ICD-9 code of K51; ICD-10 code of E11; or self-reported diagnosis by a doctor at ≥ 30 years of age (fields 2,443 and 2,976). Individuals with type 1 diabetes [self-reported diabetes that occurred <30 years of age or E10] or gestational diabetes [self-report (field 4,011) or O24] were excluded from both cases and controls.

HDL_norm: HDL cholesterol levels were obtained from field 30760 and inverse rank normalized.

LDL_norm: field 30780. LDL cholesterol was adjusted for those individuals who reported taking one of five cholesterol lowering drugs (field 20003; Simvastatin, atorvastatin, rosuvastatin, pravastatin, Fluvastatin) by dividing the measured LDL value by 0.7^2^. The resulting value was the inverse rank normalized.

TG_norm: Triglyceride levels were obtained from field 30870 and inverse rank normalized.

CHO_norm: field 30690. Totalholesterol was adjusted for those individuals who reported taking one of five cholesterol lowering drugs (field 20003; Simvastatin, atorvastatin, rosuvastatin, pravastatin, Fluvastatin) by dividing the measured value by 0.8^2^. The resulting value was the inverse rank normalized.

Breast_caner: Cancer code field 20001 to be 1002 (breast) or ICD-10 code (field 41270, C50).

colon_sigmoid_cancer: Cancer code field 20001 to be 1022 (colon cancer/sigmoid cancer) or ICD-10 code (field 41270, C18).

female_genital_tract_cancer: Cancer code field 20001 to be 1037 (female genital tract cancer) or ICD-10 code (field 41270, C51-C58).

male_genital_tract_cancer: Cancer code field 20001 to be 1038 (male genital tract cancer) or ICD-10 code (field 41270, C60-C63).

skin_cancer: Cancer code field 20001 to be 1003 (skin_cancer) or ICD-10 code (field 41270, C43-C44).

FEV1_max_INR: Forced expiratory volume in 1-second, data field 3063. Took maximum FEV1 reads of each subject and normalized them to Z-scores.

FVC_max_INR: Forced vital capacity, data field 3062. Took maximum FVC reads of each subject and normalized them to Z-scores.

FEV1FVC_INR: Took (maximum FEV1 reads / maximum FVC reads) and normalized them to Z-scores.

Broad_depression: broadly defined depression definition^3^. Seen doctor (GP) for nerves, anxiety, tension or depression, data field 2090. Or seen a psychiatrist for nerves, anxiety, tension or depression, data field 2100. Or ICD-10 code (field 41270, F32-34, F38-F39).

Environmental covariate definition:

BMI: (field 21001) excluded women pregnant at time of recruitment (field 3140). BMI was transformed by rank-based inverse normal transformation in R.

AGE: Age at recruitment, field 21022.

Alcohol_inake_frequency: Alcohol inake frequency, field 1558. We transformed the categorical scale to integer scale: “Daily or almost daily” -> 1; “Three or four times a week” -> 2; “Once or twice a week” -> 3; “One to three times a month” -> 4. “Special occasions only” -> 5; “Never” -> 6; “Prefer not to answer” was excluded. As in <https://biobank.ndph.ox.ac.uk/ukb/coding.cgi?id=100402>.

Pm2.5: Particulate matter 2.5 air pollution 2010, field 24006.

SEX: field 31.

smoking_years: packed years of smoking, field 20161. Every subject with missing values were set to be 0.

townsendscore: Townsend deprivation index at recruitment, field 22189.

**Table S7: Genotyping and subject quality control**

|  | # variants | # variants removed in this step | # subjects | # subjects removed in this step |
| --- | --- | --- | --- | --- |
| Step 1: Initial variant QC (Array) |  |  |  |  |
| Genotyped variants | 805,426 |  |  |  |
| Autosomal variants | 784,256 | 21,170 |  |  |
| Covered by both arrays | 733,322 | 50,934 |  |  |
| Batch level qc | 687,004 | 46,318 |  |  |
| SNPs only (indels removed) | 674,489 | 12,515 |  |  |
| Step 2: Subject QC^1^ (Array) |  |  |  |  |
| Genotypes available |  |  | 488,377 |  |
| Phenotypes available |  |  | 488,282 | 95 |
| Genetic and reported sex match |  |  | 487,910 | 372 |
| Sex chromosomes non-XX XY |  |  | 487,440 | 470 |
| Outliers in heterozygosity/missing rate |  |  | 486,477 | 963 |
| "Caucasian" (f.22006) |  |  | 408,186 | 78,291 |
| Individual call rate > 99% |  |  | 366,752 | 41,434 |
| Unrelated^2^ |  |  | 307,259 | 59,493 |
| Step 3: Final variant QC2 (Array) |  |  |  |  |
| call rate > 99% | 639,862 | 34,627 | 307,259 | NA |
| HWE p<5x10-8 | 610,019 | 29,843 | 307,259 | NA |
| MAF > 5% | 320,173 | 289,846 | 307,259 | NA |
| Step 4: Merging with hp3 imputed SNPs^3^ | **966,766** |  | **307,259** | NA |

^1^Subject QC was performed using the 674,489 variants.

^2^Unrelated subjects were used for final variant QC and then the set of variants were selected for the full set of subjects.

^3^hapmap3 imputed SNPs with score > 0.8 share the same QC procedure as array SNPs: call rate > 99%, HWE p <5x10-8, MAF > 5%.

**Simulation UKBB dataset quality control**

The same set of N=276,050 subjects in the previous study^4^ were used here: UKBB subjects with self-reported European ancestry (f.22006) and not related to any other subjects in the UKBB dataset (f.22021). We took the intersection of the UKBB imputed 93 million variants, hapmap3 list variants and variants in the 1000 Genome project, with the following quality control procedure: imputation score > 0.3, call rate >99%, Hardy-Weinberg Equilibrium (HWE) p-value > 5x10^-4^, and minor allele frequency (MAF) > 0.05. This resulted in the set of 396,330 variants.

**PRS fitting methods and PRS-by-E effect linear regression**

**Table S8: GWAS summary statistics information to fit PRS**

| Trait information | Sample size | PMID |
| --- | --- | --- |
| FEV1FVC_INR | 255,647 | 38165527 |
| FEV1_max_INR | 255,647 | 38165527 |
| FVC_max_INR | 255,647 | 38165527 |
| HDL_norm | 94,288 | 24097068 |
| LDL_norm | 89,866 | 24097068 |
| TG_norm | 90,989 | 24097068 |
| T2D | 156,109 | 28566273 |
| HbA1c | 123,665 | 28898252 |
| BC | 228,951 | 29059683 |
| depression | 142,308 | 29700475 |
| CHO_norm | 94,571 | 24097068 |
| CAD | 61294 | 21378990 |
| height | 252,230 | 25282103 |

**A guidance and discussion on reference panel**

As with most methods that rely on summary statistics and an external LD reference panel, the choice of reference panel can influence performance. Methods that use partial LD information^5,6^ are relatively more robust, as they rely mainly on the diagonal elements of the squared LD matrix and thus avoid some LD noise. However, even these approaches still require that the reference and target cohorts share the same ancestry. In contrast, methods that use the full LD matrix^4,7,8^ and BV-LDER-GE are more sensitive to LD mismatch. For these methods, we recommend using a high-quality, large-sample reference panel with matched ancestry to the target dataset (e.g., UK Biobank) and being cautious when applying the method across ancestries or in low-MAF bins.

**Table S9: Comparison of the parameter meanings of the three tests**

| Test type | Parameter tested | Parameter meaning |
| --- | --- | --- |
| Test for ρ_IG_ alone | ρ_IG_ | GE genetic covariance between the additive effect and the GE interaction effect |
| Test for h_I_^2^ alone | h_I_^2^ | Proportion of phenotypic variance explained by the GE interaction effects |
| Joint test | squared Mahalanobis distance of ρ_IG_ and h_I_^2^ | The aggregated magnitude of ρ_IG_ and h_I_^2^ |

**References:**

1. Baurley, J.W. & Conti, D.V. A scalable, knowledge-based analysis framework for genetic association studies. *BMC bioinformatics* **14**, 1-10 (2013).

2. Unit, E.S. Efficacy and safety of cholesterol-lowering treatment: prospective meta-analysis of data from 90 056 participants in 14 randomised trials of statins. *Lancet* **366**, 1267-1278 (2005).

3. Howard, D.M. et al. Genome-wide association study of depression phenotypes in UK Biobank identifies variants in excitatory synaptic pathways. *Nature communications* **9**, 1470 (2018).

4. Song, S., Jiang, W., Zhang, Y., Hou, L. & Zhao, H. Leveraging LD eigenvalue regression to improve the estimation of SNP heritability and confounding inflation. *The American Journal of Human Genetics* **109**, 802-811 (2022).

5. Bulik-Sullivan, B.K. et al. LD Score regression distinguishes confounding from polygenicity in genome-wide association studies. *Nature genetics* **47**, 291-295 (2015).

6. Miao, J. et al. Reimagining Gene-Environment Interaction Analysis for Human Complex Traits. *bioRxiv*, 2022.12. 11.519973 (2022).

7. Ning, Z., Pawitan, Y. & Shen, X. High-definition likelihood inference of genetic correlations across human complex traits. *Nature genetics* **52**, 859-864 (2020).

8. Dong, Z., Jiang, W., Li, H., Dewan, A.T. & Zhao, H. LDER-GE estimates phenotypic variance component of gene-environment interactions in human complex traits accurately with GE interaction summary statistics and full LD information. *bioRxiv*, 2023.11. 22.568329 (2023).
